# Organizational participatory research: a systematic mixed studies review exposing its extra benefits and the key factors associated with them

**DOI:** 10.1186/s13012-017-0648-y

**Published:** 2017-10-10

**Authors:** Paula L. Bush, Pierre Pluye, Christine Loignon, Vera Granikov, Michael T. Wright, Jean-François Pelletier, Gillian Bartlett-Esquilant, Ann C. Macaulay, Jeannie Haggerty, Sharon Parry, Carol Repchinsky

**Affiliations:** 1 Department of Family Medicine, McGill University, 5858 Côte-des-Neiges, Suite 300, Montréal, Quebec H3S 1Z1 Canada; 2 Department of Family Medicine, Sherbrooke University, 150 Place Charles Lemoyne suite 200, Longueuil, Quebec J4K 0A8 Canada; 3 Catholic University of Applied Sciences Berlin | Institute for Social Health, Köpenicker Allee, 39-57 10318 Berlin, Germany; 4 University of Montreal Mental Health Research Institute, Montreal, Canada; 5 CIET/Participatory Research at McGill (PRAM), 5858 Cote de Neiges, 3rd floor, Montreal, QC H3S 1Z1 Canada; 6 West Island YMCA, |230 Brunswick Blvd, Pointe-Claire, Quebec H9R 5N5 Canada; 7 Special Projects, Canadian Pharmacists Association, 1785 Alta Vista Drive, Ottawa, ON K1G 3Y6 Canada

**Keywords:** Participatory research, Action research, Systematic review

## Abstract

**Background:**

In health, organizational participatory research (OPR) refers to health organization members participating in research decisions, with university researchers, throughout a study. This non-academic partner contribution to the research may take the form of consultation or co-construction. A drawback of OPR is that it requires more time from all those involved, compared to non-participatory research approaches; thus, understanding the added value of OPR, if any, is important. Thus, we sought to assess whether the OPR approach leads to benefits beyond what could be achieved through traditional research.

**Methods:**

We identified, selected, and appraised OPR health literature, and at each stage, two team members independently reviewed and coded the literature. We used quantitative content analysis to transform textual data into reliable numerical codes and conducted a logistic regression to test the hypothesis that a co-construction type OPR study yields extra benefits with a greater likelihood than consultation-type OPR studies.

**Results:**

From 8873 abstracts and 992 full text papers, we distilled a sample of 107 OPR studies. We found no difference between the type of organization members’ participation and the likelihood of exhibiting an extra benefit. However, the likelihood of an OPR study exhibiting at least one extra benefit is quadrupled when the impetus for the study comes from the organization, rather than the university researcher(s), or the organization and the university researcher(s) together (OR = 4.11, CI = 1.12–14.01). We also defined five types of extra benefits.

**Conclusions:**

This review describes the types of extra benefits OPR can yield and suggests these benefits may occur if the organization initiates the OPR. Further, this review exposes a need for OPR authors to more clearly describe the type of non-academic partner participation in key research decisions throughout the study. Detailed descriptions will benefit others conducting OPR and allow for a re-examination of the relationship between participation and extra benefits in future reviews.

**Electronic supplementary material:**

The online version of this article (10.1186/s13012-017-0648-y) contains supplementary material, which is available to authorized users.

## Background

Participatory research is an umbrella term for a host of collaborative approaches to research where academics conduct research with non-academic partners [1] and has been defined as a “systematic inquiry, with the collaboration of those affected by the issue being studied, for purposes of education and taking action or effecting social change” [2]. This research approach produces theoretical or evaluative knowledge, similar to conventional quantitative and qualitative methodologies, and also blends research with action, thereby producing knowledge that can inform healthcare practices, services, and organizations [3]. The origins of this family of research approaches have been referred to the Northern and Southern traditions [4]. The Northern tradition is rooted in the work of Kurt Lewin on action research in the 1940s. In his seminal paper, Lewin called for collaborations between academic researchers and practitioners to effect sustainable change, writing that action research “needs the best of what the best among us can give, and the help of everybody” [5]. Action research engages academic researchers and non-academic partners in a democratic and iterative research cycle of planning, action, and fact finding about the effect of the action [5, 6]. A central tenet of action research is that working collaboratively will lead to changes in the academics and non-academics alike; thus, the approach integrates research, action, and education [5, 6]. In the 1980s, Chris Argyris and Donald A. Schön built on this tradition with their work on organizational learning and action science [7] and reflective practice, or “kind of action research that enhances common sense, a form of inquiry that builds on and feeds back to modify what we already know-in-practice.” [8] The Southern tradition, for its part, stems from the work of Paolo Freire, in Brazil [9]. This tradition emphasizes empowerment and emancipation of marginalized or oppressed populations. Much of community-based participatory research follows from this tradition [1, 10].

The research literature demonstrates the dynamic evolution of participatory research with numerous constructive debates, methodological papers, reviews, and books regarding similarities and nuances between action research, community-based participatory research, and several related approaches such as collaborative action research, cooperative action research, participatory action research, transformative research, emancipatory action research, and so forth [11–13]. For instance, participatory research does not necessarily follow the *planning*, *action*, and *fact finding* cycle outlined by Lewin [5]. Also, what exactly does it mean to ‘engage’ or ‘collaborate’ with non-academic partners in communities vs. in organizations? Does this collaboration occur during the action phase (e.g., design or implementation of an intervention) or during the research phase (e.g., definition of the research question or data collection and analysis)? In their large systematic review of what the authors refer to as action research, Waterman et al. [3] documented non-academic partners’ participation in four stages of the research (information gathering, planning, implementation, and evaluation) according to six categories of participation (co-option, compliance, consultation, cooperation, co-learning, and collective action). Notably, they documented that these stakeholders’ category of participation often varies throughout the phases of a given study, a finding which corroborates previous work [14]. Moreover, the many labels for the various approaches of the family of collaborative research are not always used to mean the same thing [3, 11, 15]. In line with other typologies of stakeholder participation in research [14, 16, 17] (Table 1), and a previous review by co-authors on community-based participatory research [18], we submit that participatory research refers to non-academic partners participating with university researchers in at least three key research decisions: (a) identifying the research question(s); (b) setting the methodology, collecting and/or analyzing the data, or interpreting the findings/results; and (c) implementing or disseminating the research findings. All types of qualitative, quantitative, and mixed methods may be used with this research approach [19].

**Table 1:** The relationships between our conceptual framework and benchmark works on OPR: types of participation

Our framework	Waterman et al. 2001 [3]	Munn-Giddings et al. 2008 [24]	Holter & Schwartz-Barcott 1993 [17]	Corwall & Jewkes 1995 [16]	Hart & Bond 1995 [14]
Consultation: Non-academic partners are consulted by (and influence) researchers for research questions; and methodology, or collecting, analyzing, or interpreting data; and uptake or dissemination of research findings (no research co-governance).	Consultation: Local opinions asked; researchers analyze and decide course of action.	Passive participation: Providing input (information and data) for the study.	Technical collaboration: Researcher identifies problem and intervention; the goal is to gain practitioner’s interest in the research and agreement to facilitate and help with its implementation.	Shallow participation: Researchers control the entire process.	Experimental: Researcher is the expert, participants are respondents.
Co- construction: Non-academic partners work actively with researchers in determining: Research questions; and methodology, or collecting, analyzing, or interpreting data; and uptake or dissemination of research findings (research co-governance).	Cooperation: Locals, with outsiders, determine priorities; outsiders direct the process.	Active participation: Making a contribution to the research process.	Mutual collaboration: The researcher and practitioners come together to identify potential problems, their underlying causes and possible interventions.	Increasingly deep participation: A movement towards the researchers relinquishing control and devolving ownership of the process to those whom it concerns.	Organizational: Locals determine research focus and consult researcher to conduct research.
	Co-learning: locals & researchers share their knowledge, create new understanding, & jointly form action plans.				Professionalizing: Outside researcher and locals collaborate; roles are merged.
	Collective action: locals set own agenda & mobilize to carry it out without outside initiators/ facilitators.		Enhancement: Researcher as facilitator; assists practitioners to raise their collective consciousness.		Empowering: Outside researcher and locals are co-researchers and co-change agents; Roles are shared.

In health, organizational participatory research (OPR) is carried out with organizations, where an organization is a “context of action in which relationships of cooperation, exchange, and conflict between actors with divergent interests are being established and managed” [20] with rules and hierarchies, and which fluctuates in response to changes in the environment. A health organization is any organization offering health-related services such as hospital wards, primary care clinics, or long-term care facilities. In health organizations, OPR can be used to develop research capacity and reflective practice (e.g., practitioners not only collect facts regarding their practice, but reflect on their practice to uncover and understand tacit knowledge), and it produces organizational learning (i.e., practitioners not only reflect on and amend organizational activities, but also the governing strategies behind them) [7]. It is used to implement organizational changes and practice improvement by, for example, addressing challenges or solving clinical and non-clinical problems, or to develop and implement innovations or interventions [3, 21–23]. Additionally, OPR empowers health professionals and can contribute to their professional development (e.g., increased skills and knowledge in areas such as management, clinical practice, education, and research), and results in improvements in healthcare (e.g., improved patients knowledge about medication, improved infection control) and patient satisfaction [3, 15, 24, 25]. Indeed, this research approach is recommended because it “appears to have the potential to assist practitioners, managers and policy makers in their efforts to provide high-quality healthcare” [3].

To date, four literature reviews have focused on OPR in health, including studies with varying degrees of organization participation throughout the research (from cooperation to full engagement in the research and/or ensuing actions) [3, 15, 24, 25]. These reviews are each narrow in focus examining only nursing [24], adult intensive care unit settings [15], UK settings [3], or only practice-based nursing [25]. They conclude that OPR empowers health professionals and contributes to their professional development. However, the association of OPR outcomes with processes of participation remains unclear because these reviews included OPR studies without distinguishing between (or among) types of participation. Furthermore, the challenges associated with this research approach such as increased workload of organization members, time constraints and conflicting commitments, and disruption, such as resistance to change or interpersonal tensions [3, 25], further underscore the need to understand if the benefits of this approach outweigh the costs. Participatory research approaches are also a more time-consuming research approach, compared to non-participatory research [26], and may require extra effort to work through challenges, among them practitioners’ research values and skills, and communication difficulties [25]. Thus, understanding outcomes in terms of the added value of OPR, if any, is important.

### Aims and objectives

To better understand the processes and outcomes of OPR in health, we conducted a two-phase systematic review of qualitative and quantitative evidence. This manuscript pertains to the first phase which was quantitative and compared two modes of health organization participation in research decisions:

1. Co-construction: organization members work actively with university partners in at least the three phases of the research cited above (research co-governance).
2. Consultation: organization members are consulted by university partners in at least these three phases of the research, and provide input that influences the research decisions (no research co-governance).

This dichotomy is based on various frameworks of non-academic partner participation in research (Table 1). Although reductionist, we chose to use a dichotomy because research reports often lack the detail necessary to further classify the nature of non-academic partner participation [3, 24].

We sought to assess whether the OPR approach contributes to benefits over and above achieving the research objective (i.e., extra benefits). Our specific review questions were:

- What are the types of extra benefits of OPR in health; that is, research conducted in partnership between health organizations and university researchers (co-construction and consultation)?
- To what extent are these extra benefits associated with factors such as the type of participation (co-construction vs. consultation)?

Answering these questions is important to better understand OPR effects. The second phase of this review is an ongoing complementary qualitative data analysis of quantitative and qualitative evidence and aims to provide a rich detailed description of the processes and outcomes, including challenges and pitfalls, of OPR and suggest guidelines for planning and conducting OPR.

## Methodology and methods

Systematic mixed studies reviews are an emerging form of literature review that use mainly textual data to combine qualitative and quantitative evidence extracted from qualitative, quantitative and mixed methods studies [27, 28], and can provide a highly practical understanding of complex health interventions and programs [23, 29–31]. In this review, we synthesized qualitative and quantitative evidence to test the hypothesis that the likelihood a co-construction type OPR study yields extra benefits is greater than for studies using consultation type OPR, and that five other variables are influencing factors (Table 2). Our synthesis design combined quantitative content analysis method and a multivariate logistic regression.

**Table 2:** List of variables for multivariate analysis

Variables	Rationale	Values
Dependent variable		
Extra benefit (yes/no) (raw kappa^c^ = 0.506^a^	Extra benefits offer possibilities for increasing understanding and action [48]	Present/absent (1/0)
Independent variables		
Participation of non-academic partners (raw kappa^c^ = 0.590^b^	Co-construction type participation of at least one non-academic partner group (i.e., nurses, staff, physicians, patients, etc.) will yield more extra benefits [3]	Co-construction/consultation (1/0)
OPR initiation (researchers/organization) (raw kappa = 0.534^b^	OPR initiated by the organization members will yield more extra benefits	Organization/Academic or joint (1/0)
Number of non-academic groups (i.e., nurses, therapists, physicians, patients, etc.) who participate in the research	A greater number of participant groups will increase the potential for unanticipated advantages [3]	Number of groups (n)
Participation of management	Participation of management will yield more extra benefits	Present/absent (1/0)
Duration of the study	Longer studies will yield more extra benefits [3]. Research indicates many partnerships due not survive their first year if they do not manage to build productive working relationships [49, 50]	More than 1 year/1 year or less (1/0)
Date of publication	Studies published subsequent to the Waterman et al. [3] (2001) systematic review will exhibit more extra benefits	2005 or later/before 2005 (1/0)

^a^ *p* < 0.001

^b^ *p* < 0.0001

^c^Extra benefit: rater 1 coded all studies as “yes” or “no”; rater 2 coded “yes”, “no”, or “unsure” (*n* = 3). When “unsure” are deleted, Kappa = 0.51, *p* < 0.001). When “unsure” are converted to “yes”, Kappa = 0.45. When “unsure” are converted to “no”, Kappa = 0.51

Participation: rater 1 coded “consultation” or “co-construction” whereas rater 2 coded “consultation”, “co-construction” or “unsure” (*n* = 4). When “unsure” are converted to “consultation”, Kappa = 0.601. When “unsure” are converted to “co-construction”, Kappa = 0.675

To help ensure our work is relevant to health organizations, we used a participatory approach, partnering with managers from a variety of health organizations (Table 3) to define our research objectives, refine our data collection and analysis, and interpret and disseminate findings. Below we present our methods in line with the PRISMA statement [32]. As per PROSPERO inclusion criteria, this review is not registered.

**Table 3:** Members of the research team

Core group	
Paula L Bush, PhD	Department of Family Medicine, McGill University, Member of CIET-PRAM (Participatory Research at McGill (PRAM); http://pram.mcgill.ca/index.php)
Pierre Pluye, MD, PhD	Department of Family Medicine, McGill University Member of CIET-PRAM, http://pram.mcgill.ca/index.php
Christine Loignon, PhD	Department of Family Medicine, University of Sherbrooke
Ann C Macaulay, CM MD FCPC FRCPC (Hon) CAHS	Department of Family Medicine, McGill University, Founding director of PRAM (http://pram.mcgill.ca/index.php)
Organization partners	
Sharon Parry, BSc	Director of a local YMCA, a charitable organization dedicated to the wellbeing of individuals and communities
Jean-François Pelletier, PhD	Director of the “*Comité de l’expertise patient-partenaire de Hôpital Louis-H. Lafontaine*”*,* an hospital-based committee including patients as partners and experts in mental health
Carol Repchinsky, BSc	Editor, Canadian Pharmacists Association (CPhA), a national organization of individual pharmacists supported by its business of publishing high quality drug and therapeutic information for healthcare professionals
Jeannie Haggerty, PhD	Director the McGill University Practice Based Research Network
Co-investigators	
Michael T. Wright, PhD LICSW MS	Co-founder of the International Collaboration for Participatory Health Research (ICPHR; http://www.icphr.org/)
Gillian Bartlett-Esquillant, PhD	Department of Family Medicine, McGill University
Health librarian	
Vera Granikov, MLIS	Research embedded health librarian; Department of Family Medicine, McGill University,

### Eligibility criteria

In line with the literature on systematic mixed studies reviews [28], we collected all types of evidence to better understand OPR and included original qualitative, or quantitative, or mixed methods empirical research. Eligible studies were reported in English or in French, health related, and conducted within a health organization using an OPR approach (either co-construction or consultation). Also, given our focus on the extra benefits of OPR, it was necessary that our included studies reported outcomes. Thus, to be eligible, studies had to report a practice change initiative authors had implemented, or attempted to implement (e.g., a revised healthcare procedure). Through an iterative process of criteria development, testing, and modification by the core team (Table 3), consultation with organization partners and co-researchers, and modification and further testing, we developed and refined our criteria definitions. Because of their complexity, some eligibility criteria could only be assessed with the full text; thus, there are additional selection (full text) criteria compared to those used at the identification stage (titles/abstracts) (Tables 4 and 5 and Additional file 1). Given that no one term is used to refer to OPR, the description of the research approach was used to determine eligibility for the OPR criterion, rather than the label used.

**Table 4:** Six identification criteria

Identification criteria (title and abstract)
1. The reference is in French or English
2. The reference reports an empirical research study (i.e., an original qualitative, or quantitative, or mixed methods study)
3. The reference concerns health-related research (i.e., deals with a health issue or health professional/organization development)
4. The reference concerns research with (or within) a health organization
5. The paper reports non-academics partnering with academic researchers in the research process in either consultation or co-construction manner
6. The reference reports a study about practice change

**Table 5:** Nine selection criteria

Selection criteria (full text paper)
1. The full text paper is available
2. The full text paper is written in English or French
3. The paper reports empirical research (i.e., an original qualitative, or quantitative, or mixed methods study)
4. The study concerns health-related research (i.e., deals with a health issue or health professional/organizational development)
5. The study concerns research with (or within) a health organization
6. The paper reports non-academics partnering with academic researchers in the research process in either consultation or co-construction manner
7. The paper reports a study where OPR is the collaborative change intervention
8. The paper reports OPR-related outcomes
9. The study includes sufficient description of the OPR process

### Information sources and search strategy

To ensure we captured relevant studies for this review, we cast a wide net, using multiple terms for participatory research and for health organizations (Additional file 2). Terms were informed from 39 studies identified as relevant to the review during preparatory work and also suggested by the research team members and the search strategy was developed by two health librarians experienced in searching for systematic reviews, and peer reviewed by three other specialized librarians. Moreover, the strategy was tested to ensure it captured the studies identified as relevant during preparatory work. The following databases were searched: MEDLINE including In-Process & Other Non-Indexed Citations (1946 to November 28 2012, searched using the PubMed interface), CINAHL (1981 to November 29 2012, searched using the EBSCOhost interface), Embase Classic + Embase (1947 to November 28, 2012, searched using the Ovid interface), PsycINFO (1987 to November Week 3 2012, searched using the Ovid interface), the Cochrane Library (1997 to November 29 2012), Social Work Abstracts (1968 to September 2012, searched using the Ovid interface) and Business Source Complete (1886 to November 29 2012, searched using the EBSCOhost interface). The search strategy for MEDLINE is presented in Additional file 2. Strategies for other databases are available upon request. We also searched for additional studies, theses, and conference proceedings in ProQuest Dissertations & Theses (Full Text: Health & Medicine) database, The New York Academy of Medicine – Gray Literature Report, OpenGrey, and Google. In total, 13,837 records were identified through the database search and exported to an EndNote database where duplicate references were removed. An additional 150 records were identified through forward citation tracking conducted up to June 2014. The majority of duplicates were removed using Endnote Bibliographic software. Duplicates that remained were identified and removed during the selection phase. A total of 8873 unique records were included for screening.

### Selection processes

To reduce the possibility of discarding relevant studies, we followed conventional guidance for systematic reviews [28]. Two independent reviewers (VG & PLB) began reading titles and abstracts in December 2012, coding each identification criterion as “1” for “yes,” “0,” for “no,” and “2” for “unsure.” To determine agreement regarding studies to move to the next phase, these reviewers met to discuss divergent codes. In instances where one reviewer had coded “unsure,” we used the inclusion/exclusion code of the other reviewer. When both reviewers were unsure, the abstract was moved to the next (full text) stage. When reviewers disagreed, discussions pertained to the codes, more so than to the abstracts.

The same two reviewers (VG & PLB) read the full text papers, coded the selection criteria and included papers, or discussed and resolved disagreements in the same manner as for the previous stage. It should be noted that at this stage, the two reviewers scrutinized full text papers to code them for the participation variable: “0” for no participation, “1” for consultation type participation, “2” for co-construction type participation, or “3” for unclear/unsure. Only studies reporting participation in such a way that they could be categorized as “consultation” or “co-construction” were included.

### Critical appraisal of included studies

Quality appraisal is a core component of systematic reviews [33]. Given our sample included qualitative, quantitative, and mixed methods studies, we used the *Mixed Methods Appraisal Tool*, which has been content validated and tested for reliability [28, 34]. Again, this phase was conducted by two independent reviewers (RQS and VK) with disagreements resolved by a third party. No studies were excluded based on their quality, but this appraisal is included in the textual description of each included study (Additional file 3: Table S1).

Because we were not synthesizing the research outcomes reported by the authors, but rather outcomes the authors described as being associated with the participation processes, we also appraised studies according to the description of these processes. For each of the selected studies, one reviewer extracted all text passages describing either a research participation process, a research participation outcome, or a research participation process linked with a research participation outcome. Text passages were extracted from the papers in the order they appeared and copied into excel documents (one per study). All excel documents were reviewed by the first author to ensure accuracy of extracted text passages and their categorization as research participation-related processes and/or outcomes. Only studies with clearly linked process-outcome text passages were retained for the final sample of studies included in this review.

### Data extraction and quantitative content analysis

We designed and piloted our data extraction forms (Additional file 4) and two independent reviewers extracted data from all included studies. As per quantitative content analysis [35], data were assigned numerical codes (variable values). Data pertain to four study aspects: (a) descriptive data about the study (duration, health domain and year of publication); (b) descriptive data about the research partners (academic partners’ fields of expertise, type and number of health organizations, type and number of organization partners including clinicians, managers and patients); (c) descriptive data about the initiation and type of participation; and (d) process and outcome text passages (as described above for appraisal phase). The quantitative data extracted for study aspects (a) and (b) were factual; to ensure accuracy, the lead author revised the numeric codes of the two independent reviewers and corrected discrepancies by returning to the full text papers. Data regarding the duration of the study or the different types of organization partners were missing for 65 (61%) of the included studies; thus, we sought to obtain the missing data directly from the authors. Unable to obtain author contact information for 13 (20%) studies, we emailed authors of 52 (80%) studies and received 26 (40%) responses (15 (23%) emails bounced back and 11 (17%) authors did not respond. With regard to study aspect (c), two independent reviewers coded full text articles and an inter-rater reliability score was calculated; then, reviewers discussed and corrected discrepancies by returning to the papers, and remaining disagreements were resolved by a third party. Regarding study aspect (d), for each included study, the process- and outcome-related text passages were extracted, into Excel, by one researcher and cross-checked by the lead author.

To generate the extra-benefits variable, two independent reviewers (RES & PLB) read each of the 107 abovementioned process-outcome Excel documents, and coded each extract for absence or presence of an extra benefit using a coding manual (code definitions and key examples). Then, an inter-rater reliability score was calculated. In mixed methods, this data transformation process is referred to as “quantitizing” [28, 36, 37]. In addition, two independent reviewers further coded the text passages according to types of extra benefits, which were developed using a content analysis technique (Table 6) and a capacity building framework [38]. Table 7 presents our detailed definition of these “extra benefits.”

**Table 6:** Quantitative content analysis

OPR systematic mixed studies review: 11-step coding process
1. Research team members asked to reach consensus on a codebook
2. Coders trained using a purposeful sample of documents (studies)
3. Codebook pilot tested using a random sample of 10% of documents
4. Codebook revised accordingly
5. Coding of all documents by two independent coders (assignment of excerpts of QUAL findings and QUAN results to codes using the codebook)
6. Disagreements between coders resolved by a third party
7. For each code, inter-coder agreement and reliability (kappa) score calculated
8. Preliminary findings discussed with research team members
9. Emerging categories discussed and creation of new codes when needed
10. All documents re-coded (steps 5 to 9) using new codes to increase consistency
11. Statistical analysis

**Table 7:** Definition of “extra benefits”

EXTRA BENEFITS *for the organization, staff and health professionals, patients, family, and/or caregivers, or the academic researchers.*
Extra benefits are positive outcomes that *clearly* do not meet the specific participatory research project *change* objective(s).
• Outcomes are changes that occur as a result of the participatory research project. These changes may affect the university researchers, organization members, patients or family members/carers, or the organization as a whole.
• Outcomes of interest are those associated with the participatory process.
Regarding sustainability of outcomes:
• Should the change objective be met and authors indicate that this change was maintained, this is an *anticipated* outcome, not an “extra” benefit of the OPR process. (The assumption is that no change process would have been undertaken had the objective not been for the change to be permanent.)
• Should the change objective be met and then transferred to another department/organization, this is *an extra benefit* (unless transfer was part of the change objective).
• Should the change *process* be maintained (e.g., action research group decides to continue their monthly meetings; organization members decide to do additional research), this is *an extra benefit* (unless the change objective was to implement regular meetings or a research culture).
Regarding a change in the study focus:
• In some studies, the aim of the project changes during the initial stages of the participatory process. Such changes are expected in participatory research, thus, for our purposes, the new aim will be the one we use to determine if subsequent outcomes are *extra benefits* or not.

### Synthesis

#### Quantitative analyses

##### Dependent variable

Extra benefits: Previous research suggests community-based participatory research projects exhibit a variety of positive, yet unanticipated, outcomes [18, 39]. For example, Jagosh et al. [18] illustrated how participatory research generates systemic changes and new unanticipated projects and activity. Yet, the association between such extra benefits and the participatory research process has not been measured. Therefore, we chose to examine this relationship according to the absence or presence of extra benefits.

##### Independent variables

Participation: previous systematic reviews in the area noted the difficulty determining the degree of participation required to achieve project success. Moreover, some had difficulty applying detailed frameworks of participation as articles were often lacking detail, and the level of non-academic partner participation varied within studies [3, 25]. Therefore, similar to the action research literature review of Munn-Giddings, McVicar [24], we used a dichotomy of participation, but based our operationalization of the two types of participation on previous benchmarking works (Table 1).

Duration of study: It typically takes 1 year for a partnership to develop and become fully functional [3]. Given the centrality of the partnership in OPR, we hypothesized that projects lasting 1 year or longer would be more likely to yield extra benefits compared to those lasting less than 1 year. This hypothesis is further supported by a review of community-based participatory research literature [18, 39].

Initiator of the research: In OPR, it is important that all partners agree on the importance of the research focus [2, 40]. It may be easier to generate buy-in from organization members if the impetus for the study comes from the organization. To examine this, we included this variable in the regression model, hypothesizing that the likelihood of observing extra benefits would be higher for studies initiated by the organization partners, rather than the university ones.

Managers included in the partnership: Health organizations are professional bureaucracies with a multi-dimensional hierarchy among and within service providers (physicians, pharmacists, nurses, social workers and allied practitioners) and support staff (management, material supplies, food and maintenance) [20, 41]. It follows therefore, that the practice changes sought through the research must be approved by managers in the organization. Assuming this would be smoother if managers were part of the research decision making process, we hypothesized that their participation would increase the likelihood of the OPR yielding extra benefits.

Study published in 2005 or later: The extensive systematic review by Waterman et al. [3] was the first regarding action research with health organizations. We hypothesized that subsequent OPR would have learned from this review and that this may be observable through a higher likelihood of extra benefits. To account for the lag between study design and study publication, we compared studies published in 2005 or later to those published before 2005. This choice was further supported by the fact that the median year of publication of our included studies was 2005.

Number of types of organizational partners: Given that organizational practices may involve a variety of practitioners and staff, modifying these practices could be enhanced and more successful if all stakeholder types are involved in the change process [40]. Thus, we hypothesized that a greater number of types of organizational partners would increase the likelihood of extra benefits. This was a continuous variable (range 1–9).

### Statistical methods

The inter-rater reliability for the extraction and coding of three variables (Table 2) was estimated using Cohen’s Kappa statistic [42]. For two variables (presence/absence of extra-benefits and type of participation), there was an imbalance of rater categories (e.g., one rater used the “unsure” code while the other did not), we thus calculated the raw Kappa statistic and also that of various scenarios and report all kappa values, herein. The dependent and independent variables were summarized using descriptive statistics with counts and percentages. Bivariate statistics were calculated for each independent variable using chi-square test with reported *p* values with an alpha of 0.05 to indicate statistical significance between studies with extra benefits and those without. The impact of the independent variables (participation, study duration, research initiator, presence or absence of managers, publication before or after 2005, and number of organization partner types) on the outcome of presence or absence of extra benefits was modeled using multivariate logistic regression to calculated adjusted odds ratios with 95% confidence intervals. A restricted logistic regression analysis model was used to assess the impact of the independent variables on the types of extra benefits. All statistical analyses were conducted using Statistical Analysis System Institute Inc. Software (SAS 9.4, Cary, NC, USA).

## Results

**Fig. 1:**
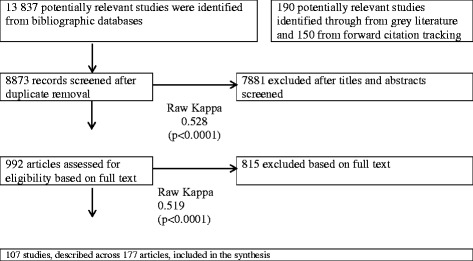
Flow diagram

The search of the peer-reviewed and gray literature led to the retrieval of 8873 unique records. Based on our aforementioned eligibility criteria, we identified 992 potentially relevant articles, 140 of which we selected for further appraisal. At this stage, we excluded an additional 33 studies due to a lack of clear description of the link between participatory processes and outcomes. Owing to the fact that several studies are described across multiple publications, our final sample of 107 studies consists of 177 publications. The flow diagram is presented in Fig. 1. The raw kappa values for the extra benefits, participation, and research initiator variables were 0.506, 0.590, and 0.534, respectively. These coefficients all indicate moderate agreement [42].

Most (*n* = 83; 77.6%) of the included studies represent co-construction type participation in research decisions. Nurses were the organization members most commonly involved as research partners (*n* = 77 studies; 30.7%). Other partners included physicians (*n* = 31; 12.4%), support staff (*n* = 13; 5.2%), and managers (*n* = 35; 13.9%). Many studies (*n* = 22; 20.6%) included more than one type of organizational member. The means and standard deviations for number of organization groups in the studies with no extra benefit or one or more extra benefits were 2.4(1.8) and 2.2(1.6) respectively (*p* = 0.66). Patients and their family or caregivers were research partners in only 15 (13.6%) and eight (7.3%) studies, respectively (Additional file 3: Table S1). We were able to determine the project duration for 89 studies. Among these, equivalent proportions of co-construction and consultation studies lasted 1 year or longer (65.3%; 47/72 and 64.7%; 11/17 respectively). No significant association was found between project duration and presence of extra benefits (Table 8).

**Table 8:** The association between extra benefits and independent factors

	0 extra benefits	≥ 1 extra benefit	Total	*p* value
	*N* (%)	*N* (%)	*N* (%)	
Type of organization member participation				
Co-construction	25 (23)	58 (54)	83 (78)	0.15
Consultation	11 (10)	13 (12)	24 (22)	
Manager in team				
Yes	9 (8)	24 (22)	33 (31)	0.36
No	27 (25)	47 (44)	74 (69)	
Published				
In 2004 or earlier	13 (12)	26 (24)	39 (36)	0.96
In 2005 or later	23 (22)	45 (42)	68 (64)	
Initiated by organization				
Yes	4 (4)	23 (22)	27 (26)	0.02*
No	32 (30)	48 (44)	79 (74)	
Duration				
Shorter than 1 year	12 (11)	19 (18)	31 (29)	0.48
One year or longer	24 (22)	52 (49)	76 (71)	

*Fisher’s exact test

Regarding the settings, nearly two thirds of studies took place in hospitals or hospital wards (*n* = 66; 61.7%). Other settings include nursing homes, primary care clinics, specialized treatment facilities, pharmacies, and community health centers. For the most part, this type of research has been conducted in the UK (*n* = 38; 35.5%), Australia (*n* = 22; 20.6%), and the USA (*n* = 14; 13.1%). The majority of the included studies used only qualitative methods (*n* = 89; 83.2). Quantitative and mixed methods were used in four (3.7%) and 14 (13.1%) studies, respectively. Additional file 3: Table S1 presents the full description of all studies in the review sample.

Two thirds (*n* = 70; 65.4%) of included studies reported at least one extra benefit of OPR. The only variable in the model that was significantly associated with extra benefits was the study initiation variable (Table 8). Contrary to our hypothesis, the logistic regression revealed no difference between the type of organization members’ participation (co-construction vs. consultation) and the likelihood of exhibiting an extra benefit. However, analyses revealed that the likelihood of a co-construction or a consultation study exhibiting at least one extra benefit is quadrupled when the impetus for the study comes from the organization, as opposed to the university researcher, or the organization and the university researcher together (OR = 4.11, CI = 1.12–14.01; Table 9).

**Table 9:** Odds ratio (OR) estimates for at least one extra benefit

Effect	OR	95% confidence limits
Co-construction compared to consultation	1.99	0.75–5.33
Project duration ≤ 1 year compared to > 1 year	1.40	0.55–3.54
Project initiated by organization compared to academic or joint initiation	4.11	1.21–14.01
Management was part of the team compared to no management	1.79	0.62–5.14
Article published in or after 2005 compared to published before 2005	2.15	0.73–6.34
Number of types of organizations groups involved	0.91	0.69–1.20

**Fig. 2:**
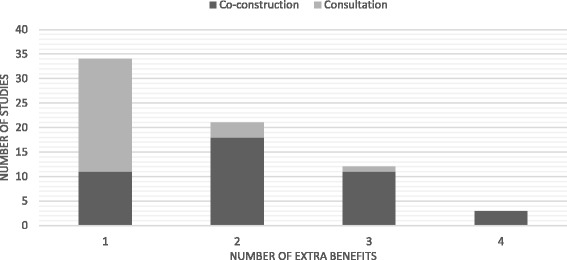
Distribution of number of extra benefits for two modes of participation

The sub-analysis defined five broad types of extra benefits. First, organization members exhibited leadership development or improvement (specifically, autonomy and confidence) exemplified in such things as empowerment/emancipation; becoming aware of skills, knowledge, and/or power; feeling ownership; advocacy; and confidence in role, skills, and/or power. Second, general workforce development was observed in terms of (a) reflective practice (e.g., improved understanding of the rationale behind certain tasks; more creative or critical evaluation of practice; increased awareness of patient needs), (b) development and/or use of new skills or tools (e.g., professional, problem solving, and/or research skills), (c) personal development (e.g., enroll in graduate school, job promotion), (d) positive changes in relations with service users (e.g., more patient centered care, learning from service users); and (e) new understandings about workplace, conditions that shape practice, and contributions of colleagues. Third, group benefits were observed in the form of improved collaborations, relations and communication among organization members, as well as staff learning from one another. Fourth, broad systemic developments or changes were observed. For example, the intended practice changes often extended beyond the target setting (e.g., transferred from the initial hospital ward to the whole hospital) or beyond the intended time frame (e.g., researchers and organization members continue to work together on subsequent OPR projects). Finally, in some studies, extra benefits were noted for the university and/or service user partners. Table 10 presents the distribution of extra benefits observed, together with specific examples from included studies. Figure 2 illustrates the distribution according to mode of participation. It is noteworthy that the majority of studies with two to four types of extra benefits are co-construction (32/36; 88.9%), whereas the majority of studies with one type of extra benefit represent consultation type participation (23/34; 67.6%). Owing to small sample sizes, no further analysis was possible.

**Table 10:** Number of studies exhibiting each of the five types of extra benefits, with examples

Type of extra benefit (number of studies)	Example (text excerpts from included studies)
Leadership (21)	For some, the process led to a greater confidence (“We do not sit back so much anymore. We speak up”) and more assertiveness (“People were starting to play with in a little bit -- try it out and feel that they had the backing”). This led to: *Greater clarity in what everyone needs, “I need time to think about that.” “I need to have it written down to understand it,” “I need to see a picture” … “I need attention.” Which helps me very much; then it is easier for me to say, “This is what I need. This is what’s helpful.”* One person noted that the group on occasion may not have listened enough to one member who would have preferred a different approach, and it might have helped to inquire about “commitment” or take a “look at alternatives.” [51]
General workforce development (41)	a Co-researchers felt able to share their ideas, the gaps in their knowledge, and recognized the importance of time for thinking and reflecting on nursing research and practice [52]
	b By allowing the client group to fully participate in the change process, new skills have been developed. These skills include team problem identification, decision making, cooperation, and in some cases leadership. With the collaborative climate being reinforced, members of the client group appear willing to take more risks in making suggestions, confronting issues, and encouraging and supporting others [53].
	c Each of the co-researchers demonstrated ongoing positive and painful enlightenment through their own personal development and participation in the action learning sets [54].
	d The members of the core group noticed a shift in their own way of thinking about patients, and in the actions of the expert patients. [55]
	e … by having the opportunity to share experiences from practice, the FARG members became more familiar with the contributions their colleagues, from other occupational groups, made to resident care. For example, an enrolled nurse member reported that as a consequence of her participation in the group she had “a bit more of an understanding about what each [staff] area gets up to [and] what challenges they have.” Similarly, another enrolled nurse member reflected on her new understanding of the different contributions that other staff members make to the care of residents when she noted, “It’s certainly opened my eyes a lot.” [52]
Group benefits (27)	The single most important indicator of full achievement of outcomes was that the work group members developed mutually supportive and trusting relationships between themselves and with the facilitator. [56]
	Data from the participants indicated an overall positive response towards action research methodology. Positive aspects of participation in the CBAR as identified by the nurses were: A feeling of teamwork; Recognition of the value of participant’s knowledge and experiences [57].
	Towards the end of the study the health professionals from both practices reported being much clearer about the nature of prediabetes and the associated risks, and placed more importance on acting systematically as a team to address the problem. [58]
	Having more meetings in itself was not enough. The nature of the communication and type of interaction was also important. People engaged with each other in a manner that was respectful, appreciative, built trust and included social bonding. Doctors and nurses often embarked on real relationships for the first time [59]
Broad systemic developments or changes (29)	The DSU nurses were able to focus activities directly related to the needs of the patients undergoing complex day surgery. Most significantly, the team members took responsibility for decisions made regarding changes and the outcomes. As a result of the opportunity to communicate openly with others, in addition to the team’s ability to think and discuss their work critically, their practice became more effective, safer for patients and patient centered. These changes were apparent to others, and provided a model of enablement that is now used elsewhere in the organization [60].
	The broad impact of the program has been confirmed by trainees from other Middle Eastern countries, who stated that they would now have the knowledge and skills to help children in pain when they returned to their home hospitals [61].
	The next step for these NCs is to further develop the research aspects of their roles. For some, this may mean handing over of part of clinical and consultancy work to create “space” for effective research. For others, it means doing other aspects of the role differently to make research happen. Their influence continues to extend beyond the organization to influence national and international healthcare agendas [62].
	There was also evidence at both teams that the changes that had occurred were part of a process that would not now easily be reversed. On the contrary, they were part of an ongoing process that now had increased momentum within the teams and their wider organizations [63].
University partners’ capacity (6)	With regard to my own empowerment I found the experience of collaboration, reflection and discussion with other participants enhanced my self-awareness, increased my appreciation for and understanding of other participants and brought me marginally closer to being able to achieve the “interpersonal elegance” for which I was striving [64].
	Finally, in terms of my own work, I have just been invited to engage in a two–year practice development partnership with a new mental health occupational therapy Trust. The plan we have negotiated is to implement a similar process as used within this study across a much larger service. This will provide an opportunity to further test and refine the approaches and conceptual frameworks developed during this inquiry [65].

## Discussion

Our results indicate that when the impetus of the OPR (consultation or co-construction) comes from the organization, the likelihood of the study resulting in at least one extra benefit is quadrupled. Although the confidence interval was fairly large, possibly due to the moderate agreement between coders, this may suggest that, where extra benefits are concerned, it is not the degree of organization members’ participation in research decisions (consultation or co-construction) that makes the difference, but rather their participation up-front. It may therefore be important for academic researchers to be open to modifying their OPR objectives to align with those of their organization partners. Moreover, it may be important for researchers to establish relationships with organizations pertinent to their research program (that is, organizations with a common interest) such that organizations’ questions may emerge from an existing relationship. Indeed, in their study of partnered-research grant applicants submitted to The Canadian Institutes of Health Research (CIHR) Sibbald, Tetroe [43] found that 35% (*n* = 17) of respondents indicated their partnerships grew from a common interest, and over 75% had established relationships.

Beckett et al. [44] found that clinicians contemplating participation in clinical research studies follow decision making processes similar to those outlined by Prochaska and DiClemente [45] for people contemplating changes in health behaviors: pre-awareness, awareness, information-gathering, first protocol (“action” stage in [45]), and maintenance. Seen through this lens, organization members who express research needs may be at the action stage. Should they, then, complete an OPR study and benefit from it, they may progress to the “maintenance” stage, exhibited by, for example, extra benefits such as extending the reach of their study into other sectors of their organization or conducting further research (or broad systemic change as illustrated in Table 10).

With their 2001 systematic review of action research in the UK, Waterman and her colleagues concluded: “the level of participation of those being investigated (the co-researchers) can vary. The minimum level of participation needed to guarantee success is not yet known.” To build on this, we tested the hypothesis that extra benefits (a potential indicator of success) are positively and significantly associated with co-construction type participation compared to consultation, but found no association. However, it is interesting to note that the confidence interval is approaching significance even with moderate agreement between coders; perhaps with additional data, the results would be different. Additional data is contingent upon the descriptions of processes authors provide in publications. As with previous reviews [3, 24], we found publications lacking in this regard. We encourage OPR authors to clearly describe the mode and timing of non-academic partners’ participation such that future reviews of OPR may re-examine the relationship between co-construction type participation and extra benefits. Moreover, given the importance of the OPR being initiated by the organization, examining whether initiation differs between partnerships where organizations participate in research-related decisions via consultation or co-construction.

Previous reviews on the topic included studies with a variety of collaborative approaches in specific contexts, including between 21 and 62 publications [3, 15, 24, 25]. While our review provides an update, it is also more comprehensive including 107 studies from any geographic location and health setting. Our review is also more precise regarding participation; some studies included in extant reviews did not meet our participation inclusion criterion. Our focus on, and distinguish between, two precise modes of participation in health research is unique and helps to define the field. The two forms of participation we explored in this study differ with respect to research governance (with or without co-governance), but both require that organization members are involved throughout the research process. It may be this continued involvement of non-academic partners that is important, rather than the decision-making process per se. It is also possible that the consultation mode of participation is sufficient to achieve extra benefits. This may be of importance to managers who make resource allocation decisions.

Defining and describing the incidence and type of extra benefits of OPR is a significant contribution of our work. Previous reviews regarding collaborative work involving academic researchers and health professionals have documented outcomes similar to those we report herein, such as practitioners’ increased confidence [3], knowledge, awareness, empowerment [3, 15], and skills [3, 46], effective communication and collaboration among staff [15]; and continued effects at the same, or another, location [3]. Yet, these reviews do not clarify whether these outcomes were intended or not. Moreover, they do not document an association between the collaborative research process and the outcomes. We have documented five types of extra benefits (unintended), that included studies' authors link with OPR processes.

Our results underscore that an OPR approach contributes to increased capacity, a commonly cited effect of participatory research [1, 18] We have identified and documented specific outcomes according to a capacity building inspired framework [38]and illustrate the type of capacity that can be built. Most studies included in this review report at least one extra benefit of the process at the individual, group, or organization level. Moreover, our framework of extra benefits, inspired by the New South Wales capacity building framework [38], is not unlike the benefits for service users that Spector [46] identified in her review of 13 participatory research projects with health and social service professionals. She notes that through collaborative work with university partners, professionals “gain research knowledge and skills, professional development, social support, and professional relationships that can help build capacity in their agencies through training, program evaluation, and securing funding” [46].

As with previous reviews [3, 24, 25], our work indicates that published OPR in health continues to engage non-academic partners according to a co-construction mode of participation more often than a consultation mode of participation. Also common to previous reviews is the finding that most research using this approach is carried out with nurses in hospital settings in the UK [3, 15, 24, 25]. Yet, our review suggests the feasibility of undertaking partnered research in other health organizations such as pharmacies, long-term care centers, and primary care clinics, and with a variety of health professionals and other organization members. Thus, to improve the use of research findings across all health organizations and among a host of health care practitioners and other organization members, promotion of, and advocacy for, OPR may be needed to increase its uptake. Finally, while one review found that multidisciplinary groups helped improve the translation of knowledge to practice [15], we found no association between the number of types of professionals and staff and extra benefits.

According to this review and others [3, 15, 24, 25], OPR rarely involves patients and/or their family or caregivers. This is striking given that in the UK, INVOLVE was established in 1996 to support active public involvement in public health and social care research (http://www.invo.org.uk/) and that, more recently, the Patient-Centered Outcomes Research Institute (http://www.pcori.org/) and the Strategy for Patient-Oriented Research (http://www.cihr-irsc.gc.ca/e/41204.html) were implemented in the USA and in Canada, respectively. Research exploring the benefits and drawbacks, and the facilitators and barriers to partnering with patients and the public in OPR could provide a valuable contribution to the OPR literature and provide guidance to enhance future OPR.

One of the strengths of this work is our synthesis design which illustrates a novel way to complete a complex synthesis of a large number of quantitative, qualitative, and mixed methods studies. Unlike meta-regression that uses extracted factual data from primary studies, three of our variables were developed using quantitative content analysis. Although the centennial quantitative content analysis method faces validity and reliability issues (no independent objective measurement), it can overcome a limitation of meta-regression, namely missing data [47]. Combining quantitative content analysis and multivariate regression modeling is a novel aspect of our review and particularly relevant given the large number of primary studies included and the limited number of variables is examined.

## Limitations

Our results are based on what authors reported in their research publications, and our data of interest were not the research findings, but rather authors descriptions and reflections on the OPR processes they used and OPR outcomes observed. There are, thus, some limitations inherent in our study design. First, our study design assumes no extra benefit was experienced if none was reported; however, this is not necessarily true given extra benefits, as we have defined them, were not the focus of the included studies. Moreover, given the cyclical and iterative nature of OPR, intermediate, or more proximal, extra benefits may occur (e.g., improved staff relations) that may contribute to achieving the study objective. It can be difficult for stakeholders to separate interdependent benefits from each and to attribute the various changes and learnings that occur, or actions that are taken during the research to the OPR processes themselves. We sought to overcome the limitations of what is reported in studies by using a rigorous quantitative content analysis approach to define extra benefits and identify them in the included studies. Furthermore, while it is possible that some eligible studies were not picked up with our search strategy, or lost during the selection processes, the large sample size lends strength to our results.

Second, although the dichotomy of participation we used does not fully capture the nature of stakeholders’ participation throughout an OPR, it did allow us to compare two distinct levels of organization member participation. Such a comparison is otherwise impossible given it is not feasible to, experimentally, compare OPR with non-participatory forms of organizational research. Additionally, a more nuanced analysis of participation was not feasible given that authors do not often describe the fluctuating level of participation of various partners throughout the research process [3, 24]. Again, we used content analysis, excluding studies that did not clearly describe organization partners’ participation, to attempt to overcome this reporting limitation and generate a reliable participation variable. Despite the moderate inter-rater agreement for the participation, extra benefits and study initiation variables, the fact that the likelihood a study yields at least extra benefits is quadrupled when the OPR is initiated by the organization indicates this relationship warrants further exploration. We suggest future OPR publications clarify non-academic partners’ participation in the research decisions, that is, for each main research phase, describe whether organization members work actively and co-govern research *with* academic partners, or are consulted *by* academics (providing research input without research co-governance). We also suggest that publications clearly distinguish between decision-making in research-related processes vs. intervention-related processes. Clear descriptions of OPR processes and outcomes are required to improve knowledge regarding the added value of organization-university research partnerships.

## Concluding remarks

While less resource-intensive means may be used to respond to organizational issues, this review focusses on the potential extra benefits of the OPR process, providing some insight into whether the investments in time and energy are worth it. We would like to acknowledge that merely quantifying extra benefits does not do justice to the richness of OPR. Indeed, stakeholders may judge the relevance of undertaking OPR by the quality and meaning of the benefits experienced. In the second phase of this work, we will use qualitative synthesis to generate a deeper understanding of the processes of OPR and the outcomes to which they contribute.

With this unique review, we have provided a framework for reporting and assessing OPR processes and outcomes, and have suggested potential key factors associated with extra benefits of OPR that can be tested in future research. We hope this contribution may influence organizations and academics alike to embark on satisfying OPR partnerships and to report their work in such a way that analyses of OPR processes and outcomes may be completed in the future to enhance our understanding of this research approach.

## Additional files

Additional file 1: Inclusion and exclusion criteria. (DOCX 40 kb)

Additional file 2: Search strategy. (DOCX 29 kb)

Additional file 3: Data extraction form. (DOCX 217 kb)

Additional file 4: Descriptive table of included studies. (DOCX 62 kb)

## Abbreviations

CIET-PRAM: Participatory Research at McGill
CIHR: Canadian Institutes for Health Research
OPR: Organizational participatory research
