# Supplementary material for: Organizational participatory research: a systematic mixed studies review exposing its extra benefits and the key factors associated with them
Source: Implement Sci. 2017 Oct 10;12:119. doi: 10.1186/s13012-017-0648-y (PMC5634842; doi:10.1186/s13012-017-0648-y)
Supplement: Supplementary file 1 — Inclusion and exclusion criteria. (DOCX 40 kb) [file 13012_2017_648_MOESM1_ESM.docx]

# SELECTION OF RELEVANT STUDIES BASED ON FULL TEXT DOCUMENTS

The previous stage (identification) led to the retrieval of 963 full-text documents. From these, relevant studies were selected as follows: for each full-text document identified in the previous stage, two independent reviewers assigned exclusion or inclusion codes, described below, and entered their codes into an Excel table.

Reviewers met to compare their codes. For papers where reviewers’ decisions to include do not match, reviewers tried to reach consensus as follows: reviewers presented their arguments, supported by article text excerpts, for their respective initial decisions and came to an agreed understanding for inclusion/exclusion through discussion. Papers for which reviewers are unable to reach consensus were given to a third party for arbitration (Reviewers indicated the specific contentious criteria).

**INCLUSION CRITERIA**

**A study is included when:**

1. The full text paper is available
2. The study concerns health related research (i.e., deals with a health issue or health professional/organizational development),
3. The study concerns research with (or within) a health organization (see below),
4. The paper reports empirical research (i.e., an original qualitative, or quantitative, or mixed methods study; see below),
5. The paper reports non-academics partnering with academic researchers in the research process in either an active or a passive way (see below),
6. The paper reports a study where OPR is the collaborative change intervention. The study reports OPR-related outcomes (see below),
7. The study includes sufficient description of the OPR process (see below).

NB: For study sets, read all papers and code as if one paper. That is, the inclusion criteria do not need to all be found in one paper, but they must all be present in the full set of papers for the set to be included (e.g., one paper may describe the participation process, but no change outcomes; whereas, another may be the outcomes paper, but not describe the participation process).

**EXCLUSION CRITERIA**

**1. NO FULL TEXT PAPER AVAILABLE**

The record is only a conference abstract or the full text cannot be retrieved (through ILL, from author contact)

**2. THE FULL TEXT PAPER IS WRITTEN IN ENGLISH OR FRENCH**

**3. THE PAPER DOES NOT REPORT EMPIRICAL RESEARCH**

The reference does not report an empirical research study (no CLEAR methods, no results), but is a commentary, an editorial, an essay, a letter, or a review, for instance.

NB1: Not all empirical papers will have methods/analysis/results sections. Some case studies or ‘program description’ type papers may be written more like stories.

NB2: Some papers are professional papers, not research studies, and should be excluded.

NB3: Should insufficient methodological detail be provided, this is cause for exclusion

NB4: Code reviews, or other potentially relevant non-empirical papers, as ‘3a’: useful for background.

**4. THE STUDY DOES NOT CONCERN HEALTH RELATED RESEARCH**

The reference does not concern Health related research, but focuses rather, for example, education of health care professionals (in an educational setting, e.g., university or teaching hospital), health professional (e.g., nurse) recruitment/retention, or managerial issues in a health care organization. Note we will include continuing professional development (skills) and continuing medical education (knowledge) studies, as well as studies that improve nurses’ leadership skills (e.g., studies that border management issues, but pertain to the health professionals as opposed to the managers).

**5. THE STUDY DOES NOT CONCERN RESEARCH WITH (OR WITHIN) A HEALTH ORGANIZATION**

Health organization(s) are not involved in the research. According to Friedberg (1997), an organization is a “context of action in which relationships of cooperation, exchange, and conflict between actors with divergent interests are being established and managed” (p. 43). In this sense, for our study, a health organization may include, for example, a specific hospital ward, rather than a hospital as a whole. We conceive Health in accordance with the WHO's broad definition.

For our review, a health organization is an organization that offers healthcare, health promotion, and/or health education services and products, such as (a) healthcare delivery workplaces, e.g., hospital or primary care clinic, (b) community health facilities, e.g., fitness centre, (c) health professional organizations, e.g., College of Family Physicians of Canada, (d) public health agencies, e.g., Health Canada, (e) health non-governmental organizations, e.g., Doctors of the World, and (f) disease and patient groups, e.g., the Heart and Stroke Foundation.

NB: The study must clearly indicate the organisation involved as the research partner (e.g., indicate if the study is carried out with nurses of a specific hospital ward, therapists of a particular rehabilitation centre, or staff of a specific long term care facility).

**6.** **THE STUDY DOES NOT REPORT NON-ACADEMICS PARTNERING WITH ACADEMIC RESEARCHERS IN THE RESEARCH PROCESS IN EITHER AN ACTIVE OR A PASSIVE WAY**

There is no non-academic partner participation, or no academic partner participation, or the participation is not clearly described, i.e., we are not able to discern if there non-academic participation in the research process (active or passive) or not.

NB1 : The partnership must be an academic/non-academic partnership. A group of practitioners doing research in their organisation is not OPR. A group of organisations carrying out research together is not OPR. A study where health professionals/researchers partner with patients/communities is more likely to be CBPR than OPR.

NB2: Should all selection criteria be met and both coders agree the study is to be included at this phase, then coders need to reach consensus on whether the participation is active or passive.

Non-participation

Participants are not consulted about the research process, they are co-opted to the study or merely comply with researcher directives (Waterman et al., 2001).

e.g., Co-option – token representatives are chosen but have no real input or power.

e.g., Compliance – tasks are assigned, with incentives; outsiders decide agenda and direct the process.

Co-construction participation

Non-academic partners contribute to at least 2 of these 3 research phase decisions (i.e., research process co-decision making):

(1) identifying the research question(s);

(2) setting the methodology, collecting and/or analysing the data, or interpreting the findings/results;

(3) implementation or dissemination of the research findings (as with our previous work, this requirement will be loosely applied because publication often predates this phase, and is, thus, often not addressed (Jagosh et al., 2011)).

This definition is in line with the benchmarking works on OPR of Holter and Schwartz-Barcott (1993), Hart and Bond (1995), Cornwall and Jewkes (1995), and Waterman et al. (2001). It also matches the level of non-academic partner involvement for which CIHR advocates in IKT (CIHR, 2010; Strauss, Tetroe & Graham 2009).

Consultation participation

Non-academic partners are consulted, but the study is directed by the academic researchers. i.e., non-academic partners provide some input regarding a research process decision (e.g.,

local opinions asked), but outside researchers decide on a course of action (no co-governance for the research process).

NB A study in which non-academic partners fully engage in designing and implementing a practice change intervention (participatory intervention), but do not participate in the research process (active or passive) will be categorized as a non-participation study (no participatory research).

Rationale for this co-construction/consultation dichotomy

Cornwall and Jewkes (1995) stated the difference between participatory and conventional methodological frameworks lies in “who defines research problems and who generates, analyses, represents, owns and acts on the information which is sought” (p.1668). This implies that non-academic partners participate throughout the research process; however, Holter and Schwartz-Barcott (1993) assert there is great variability in participation “ranging from simple periodic participation to facilitate the implementation of the intervention to in-depth, almost continuous collaboration throughout the entire study’’ (p. 300).

Some existing reviews had difficulty applying detailed frameworks of participation as articles were often lacking detail, and the level of non-academic partner participation varied within studies (Munten, et al., 2010; Waterman, et al., 2001). Others used less well defined frameworks making it difficult to replicate their process (Munn-Giddings, et al., 2008; Soh, et al., 2011). Similar to Munn-Giddings et al. (2008), we propose a dichotomy of active and passive participation, but further operationalize these categories based on our previous systematic review of CBPR.

**6. THE PAPER DOES NOT REPORT A STUDY USING OPR AS THE COLLABORATIVE CHANGE INTERVENTION (OPR ≠ CI)**

Participatory research with a health organization (OPR) was not used as the collaborative change intervention. That is, the participatory research process is independent of the organisational change reported in the study.

Example of a study where the OPR is independent of the change (OPR ≠ CI)

Researchers and non-academic partners carry out a participatory research to assess a practice change that was not developed or implemented in a participatory way.

Example of a study where the OPR is the intended collaborative change intervention (OPR = CI)
Researchers and non-academic partners carry out a participatory research to effect a change in practitioner or organisational practices. Examples of change include: improving nursing practice, improving support for family and patients, development of recommendations/ protocols/tools, improving clinic supervision.

**7. THE PAPER DOES NOT REPORT OPR-RELATED OUTCOMES**

The study does not report outcomes associated with the OPR process. That is, the authors must report that outcomes are associated with the participatory research process.

Outcomes may affect 1 or more of 4 groups: the organization, organisation members, patients, family members, carers, or academic researchers. Outcomes may be positive, adverse, or refer to challenges encountered. The third phase of our OPR review will focus on papers reporting adverse outcomes or challenges. Therefore, we need to capture all such studies at this stage (i.e., this is not an exclusion criterion).

# NB1: For examples of types of outcomes, refer to the data extraction sheet (synthesis phase).

# NB2: outcomes reported in studies where OPR = CI can be implicitly assessed as OPR-related outcomes, EXCEPT in instances where the outcomes are recommendations or needs-assessment type outcomes (i.e., no actually implementation/action taken).

NB: A study reporting lack of success in implementing an intended change is included IF the reasons is related to the participatory research process/research partnership. This represents an adverse outcome/challenge.

**8. THE STUDY PUBLICATION(S) DO NOT INCLUDE SUFFICIENT DESCRIPTION OF THE OPR PROCESS**

The publication(s) must include at least one mention of a consequence of the OPR process, such that we can identify the specific aspect of the OPR process the authors associate with the consequence. This does not need to be a ‘result’ determined through data collection and analysis; it may be based on the judgement of the authors.

NB: For this criterion, studies were assessed by Paula (checked by Vera). It is not a factual criterion, but rather an expert opinion (value judgement).

- Sufficient description of the OPR process (enough to distinguish between active vs. passive participation and to discern if the change intervention is intertwined in the OPR process
- Sufficient description of the three components of the sequence ‘input-process-output’ (enough to construct “a story that narrates the sequence of events” leading to outcomes) (Van de Ven & Huber, 1990, p. 214).
- In other words, we need enough description on how the process of change occurred, and led to OPR outcomes, (here this refers to the way the process contributes to the outcome.
- We need enough description of how process of change occurred and how the process of change contributed to the OPR outcomes.

**9. UNSURE**

# Further discussion and consensus needed. For example, there may be contention regarding OPR = CI or OPR-related outcomes. Indicate specific reasons for confusion.

### Reliability

For each document, the presence/absence of an exclusion code will be counted 1/0 (exclusion). In case of disagreement between reviewers, a final decision will be made by the PI. Using specialized software will allow us to follow a transparent and auditable process, and to automatically calculate an inter-reviewer agreement regarding the exclusion of documents. A kappa statistic will be calculated and interpreted similar to previous stage.

**Reference**

Pitkin, R. M., Branagan, M. A., & Burmeister, L. F. (1999). Accuracy of data in abstracts of published research articles. JAMA, 281(12), 1110-1111
