# Supplementary material for: Organizational participatory research: a systematic mixed studies review exposing its extra benefits and the key factors associated with them
Source: Implement Sci. 2017 Oct 10;12:119. doi: 10.1186/s13012-017-0648-y (PMC5634842; doi:10.1186/s13012-017-0648-y)
Supplement: Supplementary file 2 — Search strategy. (DOCX 29 kb) [file 13012_2017_648_MOESM2_ESM.docx]

| **PART I. DESCRIPTION OF STUDY** (code all that apply, e.g., could code 1,4 for practice change domain; copy/paste explanatory text excerpts where relevant) | | | | | | | |
| --- | --- | --- | --- | --- | --- | --- | --- |
| \| **Project name** \| - Specify   99. No title/name \| \| --- \| --- \| \| **1^ST^ author’s surname** \| - Specify \| \| **PRO** \| 1 Co-construction  2. Consultation \| \| **Date(s) of paper(s)** \| - Specify dates of all papers in the set (e.g., 2001, 2003, 2004a, 2004b) \| \| **Project start year** \| - Specify year started   99. No info./unclear \| \| **Project duration** \| - Specify number of months - Specify dates provided if cannot specify no. months   99. No info./unclear \| \| **Project status** \| 1. Completed 2. Subsequent work planned or ongoing 3. Stopped (not completed)   3a. specify why (e.g., lack of resources) \| | \| **Steering committee or equivalent** \| 1. None 2. action research group 3. steering committee 4. advisory committee   77. Other: specify the term used  99. No information/unclear \| \| --- \| --- \| \| **Partnership agreement** \| 1. Yes (specify term used) 2. No   99. No info./unclear \| \| **Study initiation** \| 1. Researchers 2. Organisation 3. Joint   99. No info./unclear \| \| **Possible conflict of interest** \| 1. Yes 2. No   99. No info./unclear \| \|  \|  \| | | | \| **Health Domain** \| 1. General primary care 2. General secondary  care (e.g., surgical  ward, ICU) 3. Paediatric care 4. Palliative care 5. Long term care/ geriatrics 6. Mental health 7. Oncology 8. Occupational therapy 9. Nutrition 10. Osteoporosis 11. Contraception/ reproductive care 12. HIV/AIDS 13. Diabetes 14. Chronic lung disease 15. Asthma   77. Other: specify  99. No info./ unclear \| \| --- \| --- \| | \| **Practice change domains** \| 1. Improving care practice (e.g., improved team work, nursing practice, cultural awareness, general service) 2. Improving support for family/ care givers and patients 3. Improving clinic supervision 4. Recommendations/protocols/ tools development 5. Increased referrals   77. Other: specify  99. No information/unclear \| \| --- \| --- \| \| **Country**  **where the organisation is located** \| 1. USA 2. UK 3. Canada 4. Europe 5. Australia 6. India 7. Chile 8. Brazil 9. Denmark 10. Iceland 11. Switzerland 12. South Africa   77.Other: specify  99. No information/unclear \| | | |
| **PART II. DESCRIPTION OF PARTNERS** (code all that apply; copy/paste explanatory text excerpts where relevant) | | | | | |  |  |
| \| **Organisation members** \| \| \| --- \| --- \| \| Number of organisation members (the number who are involved in the PR) \| - Specify number   99. No information/unclear \| \| Are organisation members co-authors? \| 1. yes 2. no   99. No information/unclear \| \| Type of organisation members involved \| 1. Nurses 2. Physicians 3. Occupational therapists 4. Pharmacists 5. Managers 6. Midwives 7. Social workers 8. Receptionist 9. Psychologist(added June 2, 2014) 10. physiotherapist(added June 2, 2014) 11. service/support staff (added June 2, 2014) 12. counsellor (added June 2, 2014) 13. health visitor (added June 2, 2014) 14. psychiatrist(added June 2, 2014) 15. dietician (added June 4, 2014)   77. Other: please specify (e.g., multidisciplinary)  99. No information/unclear \| | | \| **Organization** \| \| \| --- \| --- \| \| Description of the health organisation: copy/type text excerpt \| \| \| Number of organizations \| - Specify number \| \| Size of organization \| 1. Small unit (e.g., hospital ward) 2. Large organisation (e.g., whole hospital, ward ≥ 100 beds)   99. No information/unclear \| \| Type of organisation \| 1. Hospital 2. Hospital Ward/unit/wing 3. Primary care clinic 4. Pharmacy 5. Community health centre 6. Nursing home/Home health care/Palliative care centre 7. Fitness centre/ymca 8. family medicine practice 9. specialised treatment facility or centre (e.g., cancer treatment, diabetes association, substance abuse inpatient facility) 10. health ministry 11. different types of orgs (e.g., 2-3 diff. types)   99. No information/unclear  77. Other: copy/type text excerpt (e.g., admiral nurse service) \| | | | |  |  |
| \| **Patients, Family, family home-care giver** \| \| \| --- \| --- \| \| Number of Patients, Family and/or Care Givers \| - Specify number \| \| 1. Patients 2. Family/care givers   77. Other: please specify  99. No information/unclear \| \| | | | \| **Academic Researcher** \| \| \| --- \| --- \| \| Number of investigators \| - Specify number \| \| Academic department of principal investigator \| 1. Nursing/ midwifery 2. Medicine/eppi 3. Occupational Therapy/Physical Therapy 4. Pharmacology/ pharmacy & health science 5. Management 6. Social/community work 7. Education 8. Health 9. Public/population health 10. Endocrinology 11. Psychology 12. Recreation & leisure   77. Other: please copy/type text excerpt into excel sheet  99. No information/unclear \| \| Researcher employed \| 1. Inside organization 2. Outside organization   99. No information/unclear \| | | | |  |
