# Supplementary material for: Organizational participatory research: a systematic mixed studies review exposing its extra benefits and the key factors associated with them
Source: Implement Sci. 2017 Oct 10;12:119. doi: 10.1186/s13012-017-0648-y (PMC5634842; doi:10.1186/s13012-017-0648-y)
Supplement: Supplementary file 3 — Data extraction form. (DOCX 217 kb) [file 13012_2017_648_MOESM3_ESM.docx]

| **First author**  **(year)**  **Country** | Participants and Settings | | | Type of OPR | | |
| --- | --- | --- | --- | --- | --- | --- |
|  | **Organization members’ Discipline (n)** | Service Users (n) | Organization  (n) | Type of Participation | Health Domain | Types of Extra Benefits (n) |
| Lauri (1981)^1^  Finland | Nurse (16) | 0 | Primary care clinic (7) | Co-construction | General primary care | 0 |
| O’Connor [^2^](#_ENREF_2) (1987)  Guatemala | Physician (--) | 0 | Hospital (1) | Co-construction | General primary care | 0 |
| Degerham[^3^](#_ENREF_3) mar (1991)  Sweden | Manager (2)  Trade union rep. (1) | 0 | Hospital (1) | Co-construction | General secondary care | 0 |
| ^4^Barker (1994)  USA | Nurse (1)  Physician (2)  Psychologist (1)  Counsellor & other types of therapists (12) | 0 | Specialised treatment facility/centre (1) | Co-construction | Substance abuse/misuse | 3 |
| [^5^](#_ENREF_5)Smith (1995)  Canada | Nurse (11) | 0 | Hospital (1) | Co-construction | Paediatric care | 2 |
| [^6^](#_ENREF_6)Waterman (1995)  England | Nurse (17)  Physician (1) | 0 |  | Co-construction | General secondary care | 2 |
| [^7^](#_ENREF_7)Bellman (1996)  England | Nurse (12) | 0 | Hospital (3) | Co-construction | Diabetes | 0 |
| [^8^](#_ENREF_8)Ely (1996)  USA | Nurse (16) | 0 | Hospital ward/unit (1) | Consultation | Paediatric care | 0 |
| [^9^](#_ENREF_9)Robinson (1996)  Australia | Nurse (--) | 0 | Hospital ward/unit/wing (1) | Co-construction | Paediatric care | 1 |
| [^10^](#_ENREF_10)Batteson (1997)  England | Management (2) | 0 | Hospital (1) | Consultation | Occupational therapy | 1 |
| [^11^](#_ENREF_11)Breda (1997)  USA | Nurse (5)  Occupational therapist (--)  Counsellor & other types of therapists (--) | 0 | Hospital ward/unit/wing (1) | Co-construction | Mental health | 3 |
| [^12^](#_ENREF_12)Jones-Baucke (1997)  USA | Nurse (--)  Manager (--) | 0 | Hospital ward/unit/wing (1) | Co-construction | General secondary care | 0 |
| [^13^](#_ENREF_13)Chenoweth (1998)  Australia | Staff (8) | Patient (16)  Family/care giver (16) | Nursing home/home health care/palliative care centre (1) | Co-construction | Long term care/geriatrics | 1 |
| [^14^](#_ENREF_14)Harris (1998)  Australia | Nurse (--)  Manager (--) | 0 | Hospital ward/unit/wing (1) | Co-construction | Chronic lung disease;  Asthma | 1 |
| [^15^](#_ENREF_15)Cowley (1999)  England | Nurse (--)  Physician (--)  Manager (--)  Public health specialist (--)  Health commissioner (--) | 0 | Nursing home/home health care/palliative care centre (1) | Consultation | Paediatric care | 1 |
| [^16^](#_ENREF_16)Galvin (1999)  England | Nurse (4)  Midwife (1)  Health visitor (1)  Manager (1)  Physician (1) | 0 | Primary care clinic (1) | Co-construction | General primary care | 0 |
| [^17^](#_ENREF_17)Gerrish (1999)  England | Nurse (2)  Manager (--) | 0 | Hospital (1) | Co-construction | 0 | 1 |
| [^18^](#_ENREF_18)Bridges (2000)  England | Inter-professional care co-ordinator (--)  Key clinical staff (--)  Managers (2) | 0 | Hospital (1) | Co-construction | Long term care/geriatrics | 0 |
| [^19^](#_ENREF_19)Heslop (2000)  Australia | Nurse (--)  Manager (--) | 0 | Hospital (1) | Consultation | Mental health | 1 |
| [^20^](#_ENREF_20)Olsen (2000)  Denmark | Nurse (4)  Physician (1)  Manager (1)  Physiotherapist (1)  Occupational therapist (1)  Social worker (1) | 0 | Hospital (1) | Co-construction | Long term care/geriatrics | 0 |
| [^21^](#_ENREF_21)Daniels (2001)  England | Nurse (3)  Physician (1)  Manager (1)  Social Worker (1)  Counsellor & other types of therapists (1)  Health visitor (1) | 0 | Primary care clinic (1) | Co-construction | Palliative care | 2 |
| [^22^](#_ENREF_22)Kelly (2001)  England | Nurse (--)  Manager (15)  Health assistant (8) | 0 | Hospital (1) | Consultation | General secondary care | 1 |
| [^23^](#_ENREF_23)Pedlar (2001)  Canada | Unspecified (4) | Patient (9) | Hospital (1) | Co-construction | Unspecified | 1 |
| [^24^](#_ENREF_24)Taylor (2001)  Australia | Nurse (12) | 0 | Hospital (1) | Co-construction | General secondary care | 0 |
| [^25^](#_ENREF_25)Atwal (2002)  England | Nurse (--)  Physician (--)  Occupational Therapist (--)  Physiotherapist (--) | 0 | Hospital ward/unit/wing (1) | Co-construction | Orthopaedics | 0 |
| [^26^](#_ENREF_26)Cook (2002)  Australia | Nurse (--) | 0 | Hospital ward/unit/wing (1) | Consultation | traumatic brain injury | 1 |
| [^27^](#_ENREF_27)Fagermoen (2002)  Norway | Nurse (10) | 0 | Hospital ward/unit/wing (1) | Co-construction | General secondary care | 1 |
| [^28^](#_ENREF_28)Lindeman (2002)  Australia | Manager (--)  Chef (1)  Kitchen assistant (1)  Cleaner/general assistant(--)  Personal care attendant (1) | 0 | Nursing home/home health care/palliative care centre (5) | Co-construction | Long term care/geriatrics | 1 |
| [^29^](#_ENREF_29)Bellman (2003)  England | Nurse (5) | 0 | Hospital (1) | Co-construction | Post-op recovery care | 2 |
| [^30^](#_ENREF_30)Mason (2003)  Canada | Nurse (--)  Physician (14)  Social worker (--)  Psychologist (--) | 0 | Hospital ward/unit/wing (1) | Co-construction | Paediatric care | 1 |
| ^31^Wallis (2003)  Australia | Nurse (--)  Physician (1)  Pharmacist (4) | 0 | Hospital ward/unit/wing (1) | Co-construction | Oncology | 1 |
| [^32^](#_ENREF_32)Ashburner (2004)  England | Nurse (46) | 0 | Nursing home for long-term care (1) | Consultation | Long term care/  geriatrics | 3 |
| [^33^](#_ENREF_33)Attharos (2004)  Thailand | Nurse (26) | Patient (41)  Family/care giver (45) | Hospital (1) | Consultation | Peadiatric care  oncology | 0 |
| [^34^](#_ENREF_34)Craig (2004)  Australia | Service/support staff (--) | 0 | Hospital ward/unit/wing (1) | Co-construction | Diabetes | 1 |
| [^35^](#_ENREF_35)Meijer (2004)  Netherlands | Pharmacist (4) | 0 | Pharmacy (4) | Consultation | Unspecified | 1 |
| [^36^](#_ENREF_36)Puoane (2004)  South Africa | Nurse (4)  Physician (1) | 0 | Hospital (2) | Co-construction | General primary care | 3 |
| [^37^](#_ENREF_37)Senesac (2004)  USA | Nurse (3) | 0 | Hospital (1) | Co-construction | General secondary care | 0 |
| [^38^](#_ENREF_38)Spalding (2004)  England | Nurse (--)  Occupational therapist (--)  Physiotherapist (--) | 0 | Hospital (1) | Co-construction | Occupational therapy | 1 |
| [^39^](#_ENREF_39)Whitehead (2004)  England | Manager (4) | 0 | Hospital (1) | Co-construction | Osteoporosis | 1 |
| [^40^](#_ENREF_40)Clemensen (2007)  Denmark | Nurse (10)  Physician (2) | Patient (2)  Family/ caregiver (1) | Hospital ward/unit/wing (1) | Co-construction | Diabetes (foot ulcer care) | 2 |
| [^41^](#_ENREF_41)Dewing (2005)  England | Nurse (--)  Manager (--) | 0 | Unspecified (1) | Co-construction | Long term care/geriatrics | 0 |
| [^42^](#_ENREF_42)Dickinson (2005)  USA | Nurse (2) | 0 | Hospital (1) | Consultation | Contraception or reproductive care/health or natal care | 0 |
| [^43^](#_ENREF_43)Hamelin Brabant (2007)  Canada | Nurse (--)  Manager (2)  Service/support staff (--)  Union representative (--)  Other (--) | 0 | Hospital (1) | Co-construction | Unspecified | 0 |
| [^44^](#_ENREF_44)Harrison (2005)  England | Nurse (--)  Manager (1)  Service/support staff (--) | Family/care giver (--) | Hospital ward/unit/wing (1) | Co-construction | Long term care/geriatrics;  Mental health | 1 |
| [^45^](#_ENREF_45)Mitchell (2005)  England | Nurse (13)  Manager (1) | 0 | Hospital ward/unit/wing (1) | Co-construction | General secondary care | 0 |
| [^46^](#_ENREF_46)Munn-Giddings (2005)  England | Manager (--)  Front line practitioners (--) | 0 | Hospital (2) | Co-construction | General secondary care | 0 |
| [^47^](#_ENREF_47)Thomas (2005)  England | Unspecified (--) | 0 | Primary care clinic (6) | Consultation | General secondary care | 0 |
| [^48^](#_ENREF_48)Williams (2005)  USA | Service/support staff (--) | 0 | Hospital ward/unit/wing (2) | Co-construction | HIV/AIDS | 1 |
| [^49^](#_ENREF_49)Williams (2005)  USA | Nurse (2)  Dietician/Nutritionist (2) | Patient (5) | Specialised treatment facility or centre (1) | Co-construction | Diabetes | 4 |
| [^50^](#_ENREF_50)Laustsen (2005)  USA | Nurse (10) | 0 | Hospital ward/unit/wing (1) | Co-construction | General secondary care | 4 |
| [^51^](#_ENREF_51)Bellchambers (2006)  Australia | Nurse (8) | 0 | Pharmacy (1) | Consultation | Contraception or reproductive care/health or natal care | 1 |
| [^52^](#_ENREF_52)Eisenberg (2006)  USA | Manager (--) | 0 | Hospital ward/unit/wing (1) | Co-construction | General primary care | 0 |
| [^53^](#_ENREF_53)Khungern (2006)  Thailand | Hospital staff (--) | 0 | Hospital (1) | Co-construction | General primary care | 2 |
| [^54^](#_ENREF_54)McKellar (2006)  Australia | Nurse (1)  Midwife (6)  Service/support staff (1) | Parents (--) | Hospital (1) | Co-construction | Contraception or reproductive care/health or natal care | 1 |
| [^55^](#_ENREF_55)Tolson (2006)  Scotland | Nurse (30) | Patients (--)  Family/care giver (--)  Combined (21) | Different types of organizations (--) | Co-construction | General primary care | 1 |
| [^56^](#_ENREF_56)Wimpenny (2006)  England | Occupational therapist (15) | 0 | Specialised treatment facility or centre (1) | Co-construction | Occupational therapy | 3 |
| [^57^](#_ENREF_57)Brooker (2007)  England | *Total of 30 across the following disciplines:  Nurse  Occupational therapist  Manager  Psychologist  Physiotherapist  Counsellor  Psychiatrist  Locksmith  Speech & language therapist  Dance and movement therapist  Drama therapist  Reverend | Family/care giver (3) | Community health centre (4) | Consultation | Long term care/geriatrics | 2 |
| [^58^](#_ENREF_58)Crilly (2007)  England | Nurse (--)  Physician (--)  Manager (--)  Hospital consultant (--)  Service support staff (--) | 0 | Hospital (1) | Co-construction | General primary care | 0 |
| [^59^](#_ENREF_59)Evans (2007)  England | Nurse (3)  Physician (3)  Psychologist (1)  Diabetes consultant (3)  Education specialist (1)  Smoking advisor (1) | 0 | Family medicine practice (2) | Co-construction | Diabetes | 2 |
| [^60^](#_ENREF_60)Khresheh (2007)  Jordan | Manager (--)  Unspecified health professional (--) | 0 | Hospital (3)  Health ministry (1) | Co-construction | Contraception or reproductive care/health or natal care | 2 |
| [^61^](#_ENREF_61)Lofman (2007)  Finland | Nurse (18-21) | 0 | Hospital (1) | Co-construction | Orthopaedics | 0 |
| [^62^](#_ENREF_62)Pinyokham (2007)  Thailand | Nurse (10)  Manager (5) | 0 | Hospital (1) | Co-construction | Chronic lung disease | 3 |
| [^63^](#_ENREF_63)Boniface (2008)  England | Occupational Therapist (16-20) | 0 | Unclear/Unspecificed (--) | Co-construction | Nutrition | 1 |
| [^64^](#_ENREF_64)Bryant (2010)  England | Nurse (--)  Occupational therapist (--)  Manager (--)  Service/Support Staff (--) | Patient (--) | Hospital ward/unit/wing (1) | Co-construction | Mental health | 1 |
| [^65^](#_ENREF_65)Daramas (2008)  Thailand | Nurse (8) | 0 | Hospital ward/unit/wing (1) | Consultation | Paediatric care | 0 |
| [^66^](#_ENREF_66)Day (2008)  Australia | Nurse (8)  Physician (--)  Health service staff (--) | 0 |  | Co-construction | Long term care/geriatrics | 3 |
| [^67^](#_ENREF_67)Finley (2008)  Jordan | Physician (7) | 0 | Hospital ward/unit/wing (1) | Co-construction | Paediatric care;  Oncology | 1 |
| [^68^](#_ENREF_68)Heyns (2008)  South Africa | Nurse (11)  Manager (2) | 0 | Hospital ward/unit/wing (1) | Co-construction | Unspecified | 3 |
| [^69^](#_ENREF_69)Hoontrakul (2008)  Thailand | Staff workers of PCU (8) | Patient (22)  Family/care giver (--) | Hospital (1) | Consultation | General primary care;  Long term care/geriatrics | 2 |
| [^70^](#_ENREF_70)Jones (2008)  England | Nurse (16)  Physician (2)  Occupational therapist (5)  Social workers (2)  Physiotherapist (6)  Ambulance service staff (--)  Dietician/nutritionist (--)  Counsellor & other types of therapists (4)  Continence service (1)  Voluntary sector (5) | 0 | Hospital ward/unit/wing (4) | Consultation | Unspecified | 0 |
| [^71^](#_ENREF_71)Kato (2008)  Japan | Nurse (2)  Caregiving staff (2) | 0 | Nursing home/home health care/palliative care centre (1) | Consultation | Long term care/geriatrics | 0 |
| [^72^](#_ENREF_72)Mash (2008)  South Africa | Nurse (1)  Physician (1)  Manager (--)  Administrative clerk (1) | 0 | Community health centre (1) | Co-construction | General primary care | 1 |
| [^73^](#_ENREF_73)Mills (2008)  Australia | Nurse (3) | 0 | Regional division of general practice (1) | Co-construction | Contraception or reproductive care/health or natal care | 1 |
| [^74^](#_ENREF_74)Mizra (2008)  USA | Manager (2) | Patient (3) | Community health centre (2) | Co-construction | Mental health | 2 |
| [^75^](#_ENREF_75)Proctor (2008)  Australia | Social worker (12) | 0 | Community health centre (1) | Co-construction | Long term care/geriatrics | 1 |
| [^76^](#_ENREF_76)Sorenson (2008)  Denmark | Pharmacist (5) | 0 | Pharmacy (--) | Co-construction | Nutrition | 2 |
| [^77^](#_ENREF_77)Vallenga (2008)  The Netherlands | Nurse (--) | 0 | Specialised treatment facility or centre (1) | Co-construction | Mental health | 3 |
| [^78^](#_ENREF_78)Andrews (2009)  Australia | Nurse (3)  Unregulated Worker (2) | 0 | Nursing home/Home health care/Palliative care centre (1) | Co-construction | Palliative Care | 0 |
| [^79^](#_ENREF_79)Bothe (2009)  Australia | Nurse (9)  Physician (--)  Pharmacist (--)  Manager (1)  Operating theatre staff (--) | 0 | Hospital ward/unit/wing (--) | Co-construction | General secondary care | 2 |
| [^80^](#_ENREF_80)Dobransky-Fasiska (2009)  USA | Manager (--) | 0 | Community-based agency (11) | Co-construction | Mental health | 3 |
| [^81^](#_ENREF_81)Jack (2009)  England | Nurse (--)  Manager (--)  Clinical staff (--) | 0 | Specialised treatment facility or centre (1) | Consultation | General secondary care | 0 |
| [^82^](#_ENREF_82)Orford (2009)  England | Nurse (1)  Physician (1)  Manager (6)  Social worker (1)  Psychologist (4)  Counsellor & other types of therapists (7) | 0 | Specialised treatment facility or centre (2) | Co-construction | Substance abuse/misuse | 1 |
| [^83^](#_ENREF_83)Smith (2009)  Australia | Nurse (--) | 0 | Hospital (1) | Consultation | Long term care/geriatrics | 2 |
| [^84^](#_ENREF_84)Arcidiacono (2010)  Italy | Nurse (2)  Physician (2)  Social Worker (5)  Psychologist (12) | 0 | Collaboration between local researchers from Rome, Naples and the Ravenna area and experts from Italy (the Universities of Rome, Naples and Bologna) and abroad (Bath and Birmingham Universities) (--) | Co-construction | Asthma | 0 |
| [^85^](#_ENREF_85)Bellman (2010)  England | Nurse (6)  Practice Facilitator (2) | Patient (22) | Hospital (1) | Co-construction | General secondary care | 0 |
| [^86^](#_ENREF_86)Blomqvist (2010)  Switzerland | Nurse (2) | Patient (4) | Hospital (1) | Co-construction | General primary care | 1 |
| [^87^](#_ENREF_87)Costa (2010)  Brazil | Nurse (5)  Physician (1)  Service support staff (4)  Counsellor & other types of therapists (1)  Dietician/nutritionist (1)  Caregiver (2) | Patient (--) | Nursing home/home health care/palliative care centre (1) | Co-construction | Long term care/geriatrics | 0 |
| [^88^](#_ENREF_88)Dewar (2010)  Scotland | Nurse (--)  Physician (--)  Allied health care professional (--) | 0 |  | Co-construction | General primary care | 2 |
| [^89^](#_ENREF_89)Ngwerume (2010)  Australia | Pharmacist (7) | 0 | Pharmacy (1) | Co-construction | Long term/geriatrics | 3 |
| [^90^](#_ENREF_90)Smith (2010)  Scotland | Nurse (5) | 0 | Hospital (1) | Co-construction | General secondary care | 2 |
| [^91^](#_ENREF_91)Beringer (2011)  England | Nurse (3)  Physician (1)  Manager and/or administrators staff (16)  Play therapist (1) | 0 | Hospital ward/unit/wing (7) | Co-construction | Paediatric care | 4 |
| [^92^](#_ENREF_92)Chen (2011)  USA | Nurse (--)  Physician (--)  Manager (--)  Members of the Division of Infectious Diseases (4) | 0 | Hospital ward/unit/wing (1) | Co-construction | HIV/AIDS | 0 |
| [^93^](#_ENREF_93)Gregory (2011)  England | Nurse (1)  Physician (1) | Public & patient involvement representative (1) | Hospital (1) | Co-construction | General secondary care | 0 |
| [^94^](#_ENREF_94)Kristensen (2011)  Denmark | Occupational therapist (25) | 0 | Different types of organizations (1) | Consultation | Occupational therapy | 1 |
| [^95^](#_ENREF_95)Malus (2011)  Canada | Nurse (1)  Physician (2)  Manager (1)  Psychologist (1)  Service/support staff (1) | Patient (7) | Primary care clinic (1) | Co-construction | General primary care | 1 |
| [^96^](#_ENREF_96)Ottman (2011)  Australia | Nurse (--)  Social worker (--) | Patient (8)  Family/care giver (6) | Community health centre (3) | Co-construction | Long term care/geriatrics | 2 |
| [^97^](#_ENREF_97)Ruhe (2011)  USA | Nurse (3)  Physician (2) | 0 | Primary care clinic (30) | Consultation | General primary care | 0 |
| [^98^](#_ENREF_98)Andrews (2012)  Australia | Nurse (6)  Extended Care Assistants(1)  Physiotherapist (10)  Lifestyle & Leisure Office (1)  Service/Support Staff (3) | 0 | Pharmacy (2) | Co-construction | Long-term care and geriatrics | 3 |
| [^99^](#_ENREF_99)Cashman (2012)  USA | Physician (2)  Pharmacist (1)  Health promotion staff (1) | 0 | Community health centre (2) | Co-construction | General primary care | 0 |
| [^100^](#_ENREF_100)Dengler (2012)  Australia | Nurse (--)  Manager (--) | 0 | Hospital ward/unit/wing (1) | Co-construction | Paediatric care;  Palliative care | 2 |
| [^101^](#_ENREF_101)Gregorowski (2012)  England | Nurse (5) | 0 | Hospital (1) | Co-construction | Paediatric care | 2 |
| [^102^](#_ENREF_102)Joyner (2012)  South Africa | Nurse (1)  Priest (1) | 0 | Primary care clinic (5) | Co-construction | Unspecified | 0 |
| [^103^](#_ENREF_103)Murphy (2012)  Ireland | Service/support staff (--) | 0 | Resident care facility (1) | Consultation | Pharmacology | 1 |
| [^104^](#_ENREF_104)Siebens (2012)  Belgium | Nurse (5)  Physician (6) | 0 | Hospital ward/unit/wing (1) | Co-construction | General primary care | 0 |
| [^105^](#_ENREF_105)Tempest et al (2012)  England | Nurse (--)  Physician (--)  Manager (--)  Psychologist (--)  Carer/support worker (--) | Patient (--)  Family/care giver (--) | Hospital ward/unit/wing (1) | Co-construction | Unspecified | 0 |
| [^106^](#_ENREF_106)Lucas (2013)  England | Nurse (--)  Manager (--)  Physiotherapist (--)  Orthopaedic consultant (--) | Patient (5) |  | Co-construction | General secondary care | 2 |
| [^107^](#_ENREF_107)Ko (2013)  USA | Nurse (--)  Physician (--)  Pharmacist (--)  Manager (--)  Service/support staff (--) | 0 | Hospital ward/unit/wing (4) | Co-construction | Unspecified | 2 |

**References**

1. Lauri S. The public health nurse as a guide in infant child-care and education. *Journal of advanced nursing.* 1981;6(4):297-303.

2. O'Connor P, Franklin RR, Behrhorst CH. Hospital record studies as a tool for staff education: A participatory research project in Guatemala. *Journal of Community Health.* 1987;12(2-3):92-107.

3. Degerhammar M, Wade B. The introduction of a new system of care delivery into a surgical ward in Sweden. *International Journal of Nursing Studies.* 1991;28(4):325-336.

4. Barker SB, Barker RT. Managing change in an interdisciplinary inpatient unit: An action research approach. *Journal of Mental Health Administration.* 1994;21(1):80-91.

5. Smith SE. *Dancing with conflict: public health nurses in participatory action-research*, UNIVERSITY OF CALGARY (CANADA); 1995.

6. Waterman H, Grabham J. Ophthalmic theatre nursing. Part 2: frameworks for practice. *British Journal of Theatre Nursing.* 1995;5(3):5.

7. Bellman LM. Changing nursing practice through reflection on the Roper, Logan and Tierney model: the enhancement approach to action research. *Journal of Advanced Nursing.* 1996;24(1):129-138.

8. Ely EA. *Pain management: effects of a pediatric nursing unit-based intervention program*, UNIVERSITY OF COLORADO HEALTH SCIENCES CENTER; 1996.

9. Robinson A, Miller M. Making information accessible: developing plain English discharge instructions. *Journal of Advanced Nursing.* 1996;24(3):528-535.

10. Batteson R. A strategy to improve nurse/occupational therapist communication for managing patients with splints. *British Journal of Occupational Therapy.* 1997;60(10):451-455.

11. Breda KL, Anderson MA, Hansen L, Hayes D, Pillion C, Lyon P. Enhanced nursing autonomy through participatory action research. *Nursing outlook.* 1997;45(2):76-81.

12. Jones-Baucke DL. *A qualitative study of the implementation of a system to increase nurses' use of standardized nursing languages*, UNIVERSITY OF WASHINGTON; 1997.

13. Chenoweth L, Kilstoff K. Facilitating positive changes in community dementia management through participatory action research. *International journal of nursing practice.* 1998;4(3):175-188.

14. Harris LE. *'Getting closer to a good thing?' Possibilities and limitations for empowerment in nursing: an Australian action research case study*, University of New South Wales (Australia); 1998.

15. Cowley S, Billings JR. Implementing new health visiting services through action research: an analysis of process. *Journal of advanced nursing.* 1999;30(4):965-974.

16. Galvin K, Andrewes C, Jackson D, et al. Investigating and implementing change within the primary health care nursing team. *Journal of advanced nursing.* 1999;30(1):238-247.

17. Gerrish K, Clayton J, Nolan M, Parker K, Morgan L. Promoting evidence-based practice: managing change in the assessment of pressure damage risk. *Journal of nursing management.* 1999;7(6):355-362.

18. Bridges J, Meyer J, Spilsbury K. Organisation of care for older people in A&E. *Emergency Nurse.* 2000;8(3):22-26.

19. Heslop L, Elsom S, Parker N. Improving continuity of care across psychiatric and emergency services: combining patient data within a participatory action research framework. *Journal of advanced nursing.* 2000;31(1):135-143.

20. Olsen L, Wagner L. From vision to reality: how to actualize the vision of discharging patients from a hospital, with an increased focus on prevention. *International Nursing Review.* 2000;47(3):142-156.

21. Daniels L, Linnane J. Developing a framework for primary palliative care services. *British journal of community nursing.* 2001;6(11):592-600.

22. Kelly D, Simpson S. Action research in action: reflections on a project to introduce Clinical Practice Facilitators to an acute hospital setting. *Journal of advanced nursing.* 2001;33(5):652-659.

23. Pedlar A, Hornibrook T, Haasen B. Patient focused care: Theory and practice. *Therapeutic Recreation Journal.* 2001;35(1):15-30.

24. Taylor B. Identifying and transforming dysfunctional nurse-nurse relationships through reflective practice and action research. *International journal of nursing practice.* 2001;7(6):406-413.

25. Atwal A, Caldwell K. Do multidisciplinary integrated care pathways improve interprofessional collaboration? *Scandinavian Journal of Caring Sciences.* 2002;16(4):360-367.

26. Cook R, Mayne C, Gardner G, Lawrence T. Policy development through action research: managing aggressive behaviour in patients with traumatic brain injury. *Australasian Journal of Neuroscience.* 2002;15(3):5-12.

27. Fagermoen MS, Hamilton GA, Svendsen B, Hjellup H. Partners in change: action research in action in clinical practice. *Nordic Journal of Nursing Research & Clinical Studies / Vård i Norden.* 2002;22(3):45-47.

28. Lindeman M, Smith R, Vrantsidis F, Gough J. Action research in aged care: a model for practice change and development. *Geriaction.* 2002;20(1):10-14.

29. Bellman L, Bywood C, Dale S. Advancing working and learning through critical action research: creativity and constraints. *Nurse Education in Practice.* 2003;3(4):186-194.

30. Mason RA. Action research: a hospital responds to domestic violence. *Healthcare management forum / Canadian College of Health Service Executives = Forum gestion des soins de sante / College canadien des directeurs de services de sante.* 2003;16(3):18-22.

31. Wallis M, Tyson S. Improving the nursing management of patients in a hematology/oncology day unit: An action research project. *Cancer Nursing.* 2003;26(1):75-83.

32. Ashburner C, Meyer J, Johnson B, Smith C. Using Action Research to Address Loss of Personhood in a Continuing Care Setting. *Illness, Crisis, & Loss.* 2004;12(1):23-37.

33. Attharos T, Khampalikit S, Phuphaibul R, Tilokskulchai F. Development of a family-centered care model for children with cancer in a pediatric cancer unit. *Thai Journal of Nursing Research.* 2004;8(1):52-63.

34. Craig D, Seller M, Donoghue J, Mitten-Lewis S. Improving nurse management of patients with diabetes using an action research approach. *Contemporary Nurse: A Journal for the Australian Nursing Profession.* 2004;17(1-2):71-79.

35. Meijer WM, De Smit DJ, Jurgens RA, De Jong-Van Den Berg LTW. Pharmacists' role in improving awareness about folic acid: A pilot study on the process of introducing an intervention in pharmacy practice. *International Journal of Pharmacy Practice.* 2004;12(1):29-35.

36. Puoane T, Sanders D, Ashworth A, Chopra M, Strasser S, McCoy D. Improving the hospital management of malnourished children by participatory research. *International Journal for Quality in Health Care.* 2004;16(1):31-40.

37. Senesac PM. *The Roy Adaptation Model: an action research approach to the implementation of a pain management organizational change project*, Boston College, William F. Connell Graduate School of Nursing; 2004.

38. Spalding NJ. Using vignettes to assist reflection within an action research study on a preoperative education programme. *British Journal of Occupational Therapy.* 2004;67(9):388-395.

39. Whitehead D, Keast J, Montgomery V, Hayman S. A preventative health education programme for osteoporosis. *Journal of Advanced Nursing.* 2004;47(1):15-24.

40. Clemensen J, Larsen SB, Kyng M, Kirkevold M. Participatory design in health sciences: Using cooperative experimental methods in developing health services and computer technology. *Qualitative Health Research.* 2007;17(1):122-130.

41. Dewing J, Traynor V. Admiral nursing competency project: Practice development and action research. *Journal of Clinical Nursing.* 2005;14(6):695-703.

42. Dickinson A, Welch C, Ager L, Costar A. Hospital mealtimes: Action research for change? *Proceedings of the Nutrition Society.* 2005;64(3):269-275.

43. Hamelin Brabant L, Lavoie-Tremblay M, Viens C, Lefrancois L. Engaging health care workers in improving their work environment. *Journal of Nursing Management.* 2007;15(3):313-320.

44. Harrison A, Zohhadi S. Professional influences on the provision of mental health care for older people within a general hospital ward. *Journal of Psychiatric and Mental Health Nursing.* 2005;12(4):472-480.

45. Mitchell EA, Conlon AM, Armstrong M, Ryan AA. Towards rehabilitative handling in caring for patients following stroke: A participatory action research project. *Journal of Clinical Nursing.* 2005;14(3 A):3-12.

46. Munn-Giddings C, Hart C, Ramon S. A participatory approach to the promotion of well-being in the workplace: Lessons from empirical research. *International Review of Psychiatry.* 2005;17(5):409-417.

47. Thomas P, McDonnell J, McCulloch J, While A, Bosanquet N, Ferlie E. Increasing capacity for innovation in bureaucratic Primary Care Organizations: A whole system participatory action research project. *Annals of Family Medicine.* 2005;3(4):312-317.

48. Williams A, Selwyn PA, McCorkle R, Molde S, Liberti L, Katz DL. Application of community-based participatory research methods to a study of complementary medicine interventions at end of life. *Complementary Health Practice Review.* 2005;10(2):91-104.

49. Williams AS. Using participatory action research to make diabetes education accessible for people with visual impairment. *Dissertation Abstracts International: Section B: The Sciences and Engineering.* 2005;66(5-B):2883.

50. Laustsen G. Promoting ecological behavior in nurses through action research. *Communicating Nursing Research.* 2007;40:552-552.

51. Bellchambers H, McMillan M. Progressing QUM in aged care... Quality Use of Medicines. *Geriaction.* 2006;24(2):15-25.

52. Eisenberg EM, Baglia J, Pynes JE. Transforming emergency medicine through narrative: qualitative action research at a community hospital. *Health communication.* 2006;19(3):197-208.

53. Khungern J, Krairiksh M, Tassaniyom N, Sritanyarat W. Hospital quality improvement: a case study of a general hospital under the Ministry of Public Health. *Thai Journal of Nursing Research.* 2006;10(3):191-200.

54. McKellar LV, Pincombe JI, Henderson AM. Insights from Australian parents into educational experiences in the early postnatal period. *Midwifery.* 2006;22(4):356-364.

55. Tolson D, Irene S, Booth J, Kelly TB, James L. Constructing a new approach to developing evidence-based practice with nurses and older people. *Worldviews on Evidence-Based Nursing.* 2006;3(2):62-72.

56. Wimpenny K, Forsyth K, Jones C, Evans E, Colley J. Group reflective supervision: Thinking with theory to develop practice. *British Journal of Occupational Therapy.* 2006;69(9):423-428.

57. Brooker DJ, Woolley RJ. Enriching opportunities for people living with dementia: The development of a blueprint for a sustainable activity-based model. *Aging and Mental Health.* 2007;11(4):371-383.

58. Crilly T, Plant M. Reforming emergency care: Primary care trust power in action research. *Health Services Management Research.* 2007;20(1):37-47.

59. Evans PH, Greaves C, Winder R, Fearn-Smith J, Campbell JL. Development of an educational 'toolkit' for health professionals and their patients with prediabetes: The WAKEUP study (Ways of Addressing Knowledge Education and Understanding in Pre-diabetes). *Diabetic Medicine.* 2007;24(7):770-777.

60. Khresheh R, Barclay L. Practice-research engagement (PRE): Jordanian experience in three Ministry of Health hospitals. *Action Research.* 2007;5(2):123-138.

61. Lofman P, Pietila AM, Haggman-Laitila A. Self-evaluation and peer review - An example of action research in promoting self-determination of patients with rheumatoid arthritis. *Journal of Clinical Nursing.* 2007;16(3 A):84-94.

62. Pinyokham N, Fongkaew W, Chanprasit C, Simpson T. Development of a program for enhancing nurses' capacity to wean patients from mechanical ventilation. *Thai Journal of Nursing Research.* 2007;11(4):281-293.

63. Boniface G, Fedden T, Hurst H, et al. Using theory to underpin an integrated occupational therapy service through the Canadian model of occupational performance. *British Journal of Occupational Therapy.* 2008;71(12):531-539.

64. Bryant W, Vacher G, Beresford P, McKay E. The modernisation of mental health day services: Participatory action research exploring social networking. *Mental Health Review Journal.* 2010;15(3):11-21.

65. Daramas T, Chontawan R, Yenbut J, Wittayasooporn J, Nantachaipan P. Enhancing nursing practice in developmental care for preterm infants. *Thai Journal of Nursing Research.* 2008;12(2):83-93.

66. Day J, Higgins I, Koch T. Delirium and older people: What are the constraints to best practice in acute care? *International Journal of Older People Nursing.* 2008;3(3):170-177.

67. Finley G, Forgeron P, Arnaout M. Action research: Developing a pediatric cancer pain program in Jordan. *Journal of Pain and Symptom Management.* 2008;35(4):447-454.

68. Heyns T. *A journey towards emancipatory practice development*, University of South Africa (South Africa); 2008.

69. Hoontrakul D, Sritanyarat W, Nuntaboot K, Premgamone A. Development of age-friendly primary health care: case study of one primary care unit. *Thai Journal of Nursing Research.* 2008;12(2):131-140.

70. Jones SP, Auton MF, Burton CR, Watkins CL. Engaging service users in the development of stroke services: An action research study. *Journal of Clinical Nursing.* 2008;17(10):1270-1279.

71. Kato M, Izumi K, Shirai S, et al. Development of a fall prevention program for elderly Japanese people. *Nursing and Health Sciences.* 2008;10(4):281-290.

72. Mash BJ, Mayers P, Conradie H, Orayn A, Kuiper M, Marais J. How to manage organisational change and create practice teams: experiences of a South African primary care health centre. *Education for health (Abingdon, England).* 2008;21(2):132.

73. Mills J, Fitzgerald M. Renegotiating roles as part of developing collaborative practice: Australian nurses in general practice and cervical screening. *Journal of multidisciplinary healthcare.* 2008;1:35-43.

74. Mirza M, Gossett A, Chan NK, Burford L, Hammel J. Community reintegration for people with psychiatric disabilities: challenging systemic barriers to service provision and public policy through participatory action research. *Disability & Society.* 2008;23(4):323-336.

75. Proctor K, Perlesz A, Moloney B, McIlwaine F, O'Neill I. Exploring theatre of the oppressed in family therapy clinical work and supervision. *Counselling & Psychotherapy Research.* 2008;8(1):43-52.

76. Sorensen EW, Haugbolle LS. Using an action research process in pharmacy practice research-A cooperative project between university and internship pharmacies. *Research in Social and Administrative Pharmacy.* 2008;4(4):384-401.

77. Vallenga D, Grypdonck MHF, Tan FIY, Lendemeijer BHGM, Boon PAJM. Improving decision-making in caring for people with epilepsy and intellectual disability: An action research project. *Journal of Advanced Nursing.* 2008;61(3):261-272.

78. Andrews S, McInerney F, Robinson A. Realizing a palliative approach in dementia care: Strategies to facilitate aged care staff engagement in evidence-ba ed practice. *International Psychogeriatrics.* 2009;21(SUPPL. 1):S64-S68.

79. Bothe J, Donoghue J. Using action research to develop a model of patient-centred day care. *Practice Development in Health Care.* 2009;8(3):152-160.

80. Dobransky-Fasiska D, Nowalk MP, Pincus HA, et al. Public-academic partnerships: improving depression care for disadvantaged adults by partnering with non-mental health agencies. *Psychiatric services (Washington, DC).* 2010;61(2):110-112.

81. Jack BA, Littlewood C, Eve A, Murphy D, Khatri A, Ellershaw JE. Reflecting the scope and work of palliative care teams today: An action research project to modernise a national minimum data set. *Palliative Medicine.* 2009;23(1):80-86.

82. Orford J, Templeton L, Copello A, Velleman R, Ibanga A, Binnie C. Increasing the involvement of family members in alcohol and drug treatment services: The results of an action research project in two specialist agencies. *Drugs: Education, Prevention & Policy.* 2009;16(5):379-408.

83. Smith S, Lewis P, Wilson V. A 'wee' problem: using action research to facilitate a change in urine collection methods. *Neonatal, Paediatric & Child Health Nursing.* 2009;12(1):15-19.

84. Arcidiacono C, Velleman R, Procentese F. A synergy between action-research and a mixed methods design for improving services and treatment for family members of heavy alcohol and drug users. *Journal of Community & Applied Social Psychology.* 2010;20(2):95-109.

85. Bellman L, Corrigan P. Using action research to develop a thoracic support nurse role to enhance quality of care. *Nursing times.* 2010;106(22):18-21.

86. Blomqvist K, Theander E, Mowide I, Larsson V. What happens when you involve patients as experts? a participatory action research project at a renal failure unit. *Nursing Inquiry.* 2010;17(4):317-323.

87. Costa, Valcarenghi RV, Devos, Tarouco, de Almeida S, Egues. Development of a medical record for residents in a long-stay institution for the elderly. *Acta Paulista de Enfermagem.* 2010;23(6):725-731.

88. Dewar B, Mackay R. Appreciating and developing compassionate care in an acute hospital setting caring for older people. *International journal of older people nursing.* 2010;5(4):299-308.

89. Ngwerume KT, Themessl-Huber M. Using action research to develop a research aware community pharmacy team. *Action Research.* 2010;8(4):387-406.

90. Smith S, Dewar B, Pullin S, Tocher R. Relationship centred outcomes focused on compassionate care for older people within in-patient care settings. *International journal of older people nursing.* 2010;5(2):128-136.

91. Beringer AJ, Fletcher ME. Developing practice and staff: enabling improvement in care delivery through participatory action research. *Journal of child health care : for professionals working with children in the hospital and community.* 2011;15(1):59-70.

92. Chen JC, Goetz MB, Feld JE, et al. A provider participatory implementation model for HIV testing in an ED. *American Journal of Emergency Medicine.* 2011;29(4):418-426.

93. Gregory S, Poland F, Spalding NJ, Sargen K, McCulloch J, Vicary P. Multidimensional collaboration: Reflections on action research in a clinical context. *Educational Action Research.* 2011;19(3):363-378.

94. Kristensen HK, Borg T, Hounsgaard L. Facilitation of research-based evidence within occupational therapy in stroke rehabilitation. *British Journal of Occupational Therapy.* 2011;74(10):473-483.

95. Malus M, Shulha M, Granikov V, et al. A participatory approach to understanding and measuring patient satisfaction in a primary care teaching setting. *Progress in community health partnerships : research, education, and action.* 2011;5(4):417-424.

96. Ottmann G, Laragy C, Allen J, Feldman P. Coproduction in practice: Participatory action research to develop a model of community aged care. *Systemic Practice and Action Research.* 2011;24(5):413-427.

97. Ruhe MC, Bobiak SN, Litaker D, et al. Appreciative Inquiry for quality improvement in primary care practices. *Quality management in health care.* 2011;20(1):37-48.

98. Andrews S, Lea E, Haines T, et al. Reducing staff isolation and developing evidence-informed practice in the aged care environment through an action research approach to falls prevention. *Advances in Nursing Science.* 2012;35(1):3-13.

99. Cashman SB, Flanagan P, Silva MA, Candib LM. Partnering for health: collaborative leadership between a community health center and the YWCA central Massachusetts. *Journal of public health management and practice : JPHMP.* 2012;18(3):279-287.

100. Dengler KA, Wilson V, Redshaw S, Scarfe G. Appreciation of a Child's Journey: Implementation of a Cardiac Action Research Project. *Nursing research and practice.* 2012;2012:145030.

101. Gregorowski A, Brennan E, Chapman S, et al. An action research study to explore the nature of the nurse consultant role in the care of children and young people. *Journal of clinical nursing.* 2012.

102. Joyner K, Mash B. A comprehensive model for intimate partner violence in South African primary care: action research. *BMC health services research.* 2012;12(1):399.

103. Murphy K, Welford C. Agenda for the future: Enhancing autonomy for older people in residential care. *International Journal of Older People Nursing.* 2012;7(1):75-80.

104. Siebens K, Miljoen H, Geest SD, Drew B, Vrints C. Development and implementation of a critical pathway for patients with chest pain through action research. *European journal of cardiovascular nursing : journal of the Working Group on Cardiovascular Nursing of the European Society of Cardiology.* 2012.

105. Tempest S, Harries P, Kilbride C, De Souza L. To adopt is to adapt: the process of implementing the ICF with an acute stroke multidisciplinary team in England. *Disability and rehabilitation.* 2012;34(20):1686-1694.

106. Lucas B, Cox C, Perry L, Bridges J. Changing clinical team practices in preparation of patients for Total Knee Replacement: Using Social Cognitive Theory to examine outcomes of an action research study. *Orthopaedic and Trauma Nursing.* 2013;17:140-150.

107. Ko A, Leontini R, Ngian V, Hughes I, Clemson L, Chan D. Quality and safety of prescribing practices in aged care and rehabilitation units in an Australian hospital. *Asian Journal of Gerontology & Geriatrics.* 2013;8(2):68-77.
