# Supplementary material for: Organizational participatory research: a systematic mixed studies review exposing its extra benefits and the key factors associated with them
Source: Implement Sci. 2017 Oct 10;12:119. doi: 10.1186/s13012-017-0648-y (PMC5634842; doi:10.1186/s13012-017-0648-y)
Supplement: Supplementary file 4 — Descriptive table of included studies. (DOCX 62 kb) [file 13012_2017_648_MOESM4_ESM.docx]

| **Research Question** | **Parameters to address** | | |
| --- | --- | --- | --- |
| In this systematic **mixed studies** review, we will synthesize results of studies that use **participatory research** aiming to **change practice** with and within **health organizations** offering healthcare, health promotion, and health education services and products, such as (a) healthcare delivery workplaces, e.g., hospital or primary care clinic, (b) community health facilities, e.g., fitness centre, (c) health professional organizations, e.g., College of Family Physicians of Canada, (d) public health agencies, e.g., Health Canada, (e) health non-governmental organizations, e.g., Doctors of the World, and (f) disease and patient groups, e.g., the Heart and Stroke Foundation. | **Medicine**  _x_ Cochrane (CSDR, CENTRAL, etc)  _x_ MEDLINE on PubMed and/or OvidSP  ___ Global Health  _x_ EMBASE (biomedical)  _x_ CINAHL (allied health, e.g., nursing)  ___ African Index Medicus (AIM)  ___ LILACS (South American/Caribbean)  ___ POPLine (Reproductive health) | **Psychology**  _x__ PsycINFO  **Geography (Human)**  ___ PAIS International  ___ GEOBASE (part of Scopus)  **Economics**  ___ Econlit  ___ PAIS International | **Social Sciences**  ___ Science Citation Index (part of Web of Science)  ___ Social Sciences Citation Index (part of Web of Science)  ___ IBSS (International Bibliography of the Social Sciences)  _x__ Social Work Abstracts on Ovid SP  ___ Sociological Abstracts  ___ Campbell Collaboration  **Business**  _x_ Business Source Complete on EBSCO |
|  | **Grey Literature [websites, conference proceedings, etc.]**  **___**IGO Search  ___OECD iLibrary | _x_ English & French  ___ All languages  ___ Years: | **Multidisciplinary**  ___ Web of Knowledge (includes Web of Science)  _x_Scopus (includes Embase)  _x_Google Scholar |

# Database: MEDLINE on PubMed

|  | **Concept: Action research** | **Concept: Organizational setting (facilities)** | **Concept: Organizational involvement / improvement** |
| --- | --- | --- | --- |
| Thesaurus Terms / Subheadings | community based participatory research[MeSH Terms]  (community institutional relation[MeSH Terms] AND (research[Title] OR research[MeSH Terms]) | \| residential facilities[MeSH Terms]  rehabilitation centers[MeSH Terms]  physicians offices[MeSH Terms]  pharmacies[MeSH Terms]  nurseries[MeSH Terms]  hospitals[MeSH Terms]  hospital units[MeSH Terms]  fitness centers[MeSH Terms]  dental facilities[MeSH Terms]  birthing centers[MeSH Terms]  ambulatory care facilities[MeSH Terms]  academic medical centers[MeSH Terms]  health facilities[MeSH:noexp]  organizations[Mesh:noexp]  organizations, Nonprofit[Mesh:noexp] \| \| --- \| | organizational innovation[MeSH Terms] |
| Textwords | "action research" [All Fields]  "participatory research" [All Fields]  "participative research" [All Fields]  "collaborative inquiry" [All Fields]  "collaborative action" [All Fields]  "collaborative partnership" [All Fields]  "collaborative partnerships" [All Fields]  "participatory rural appraisal" [All Fields]  "participatory appraisal" [All Fields]  "emancipatory research" [All Fields]  "social reconnaissance" [All Fields]  "empowerment evaluation" [All Fields]  ((research[Title]) OR design[Title]) AND participatory[Title]  (action[Title]) AND research[Title]  CBPR [All Fields]  "community based participatory research" [All Fields]  "community based action research" [All Fields]  "participatory evaluation" [All Fields]  "community driven research" [All Fields]  "action science" [All Fields]  "cooperative inquiry" [All Fields]  "appreciative inquiry" [All Fields]  "democratic evaluation" [All Fields]  "recherche action" [All Fields] | \| organisation[Title/Abstract]  organisations[Title/Abstract]  organisational[Title/Abstract] \| \| --- \| \| organization[Title/Abstract]  organizations[Title/Abstract]  organizational[Title/Abstract] \| \| institution[Title/Abstract]  institutions[Title/Abstract]  institutional[Title/Abstract] \| \| hospital[Title/Abstract]  hospitals[Title/Abstract]  facility[Title/Abstract]  facilities[Title/Abstract]  unit[Title/Abstract]  units[Title/Abstract]  ward[Title/Abstract]  wards[Title/Abstract]  clinic[Title/Abstract]  clinics[Title/Abstract]  pharmacy[Title/Abstract]  pharmacies[Title/Abstract]  center[Title/Abstract]  centers[Title/Abstract]  centre[Title/Abstract]  centres[Title/Abstract]  team[Title/Abstract]  teams[Title/Abstract]  agency[Title/Abstract]  agencies[Title/Abstract]  association[Title/Abstract]  associations[Title/Abstract]  “care trust”[Title/Abstract])  “care trusts”[Title/Abstract]  “healthcare trust”[Title/Abstract]  “healthcare trusts”[Title/Abstract]  “service trust”[Title/Abstract]  “service trusts”[Title/Abstract]  “services trust”[Title/Abstract]  “services trusts”[Title/Abstract]  “hospital trust”[Title/Abstract]  “hospital trusts”[Title/Abstract] \| | \| ((evaluation[Title/Abstract]) OR  consultation[Title/Abstract]) AND  process[Title/Abstract]  ((management[Title/Abstract]) OR  implementation[Title/Abstract]) AND  change[Title/Abstract] \| \| --- \| \| ((management[Title/Abstract]) OR  transformative[Title/Abstract]) AND  research[Title/Abstract] \| \| "community of practice"[Title/Abstract]  "organizational learning"[Title/Abstract]  "organisational learning"[Title/Abstract]  "transformational change"[Title/Abstract] \| |

# Database: EMBASE

|  | **Concept: Action research** | **Concept: Organizational setting (facilities)** | **Concept: Organizational involvement / improvement** |
| --- | --- | --- | --- |
| Thesaurus Terms / Subheadings | exp action research/ | \|  \|  \| \| --- \| --- \|   hospital/  community hospital/  general hospital/  geriatric hospital/  exp mental hospital/  non profit hospital/  pediatric hospital/  private hospital/  public hospital/  exp teaching hospital/  health care facility/  assisted living facility/  cancer center/  community mental health center/  dental facility/  health center/  mental health center/  nursing home/  pain clinic/  pharmacy/  rehabilitation center/  residential home/  health care organization/  non profit organization/ | change management/  organization/  organizational development/  organizational efficiency/  exp planning/   \|  \|  \| \| --- \| --- \| |

| Textwords | "action research"  "participatory research"  "participative research"  "collaborative inquiry"  “collaborative action”  “collaborative partnership?”  "participatory rural appraisal"  "participatory appraisal"  "emancipatory research"  "social reconnaissance"  "empowerment evaluation"  (participatory[title] AND (research[title] OR design[title]))  (action[title] AND research[title])  “dialectical research”  “conscienti#ing research”  “participatory learning research”  “CBPR”  “community-based participatory research”  “community-based action research”  “participatory evaluation”  “participative evaluation”  “community-driven research”  “community-driven action research”  “action science”  “community-partnered action research”  “cooperative inquiry”  “dialectical inquiry”  “appreciative inquiry”  “decoloni#ing methodologies”  “democratic evaluation”  “recherche participative”  “recherche-action”  “recherche collaborative” | \|  \|  \| \| --- \| --- \|   organi#ation$.ti,ab.  institution$.ti,ab.  (hospital or hospitals).ti,ab.  (facility or facilities).ti,ab.  (unit or units).ti,ab.  (ward or wards).ti,ab.  (clinic or clinics).ti,ab.  (pharmacy or pharmacies).ti,ab.  (center or centers).ti,ab.  (centre or centres).ti,ab.  (team or teams).ti,ab.  (agency or agencies).ti,ab.  (association or associations).ti,ab.  (care trust or care trusts).ti,ab.  (healthcare trust or healthcare trusts).ti,ab.  (service? trust or service? trusts).ti,ab.  (hospital trust or hospital trusts).ti,ab. | ((evaluation or consultation or apprais$) and process).ti,ab.  ((management or implementation) and change).ti,ab.  ((management or transformative) and research).ti,ab.  (community ADJ1 practice).ti,ab.  (organi#ational learning).ti,ab.  transformational change.ti,ab.   \|  \|  \| \| --- \| --- \| |
| --- | --- | --- | --- | --- | --- | --- | --- |

# Database: CINAHL

|  | **Concept: Action Research** | **Concept: Organizational Setting** | **Concept:** |
| --- | --- | --- | --- |
| Thesaurus Terms / Subheadings | MH "Action Research"  (MH” Community-Institutional Relations”  AND (TI research OR MH  “Research”)) | (MH "Emergency Service+")  (MH "Midwifery Service+")  (MH "Nursing Service")  (MH "Obstetric Service")  (MH "Occupational Therapy Service")  (MH "Outpatient Service")  (MH "Pain Clinics")  (MH "Pharmacy Service+")  (MH "Physical Therapy Service")  (MH "Psychiatric Service")  (MH "Health Facilities")  (MH "Academic Medical Centers")  (MH "Alternative Health Facilities")  (MH "Ambulatory Care Facilities+")  (MH "Community Health Centers")  (MH "Dental Facilities+")  (MH "Fitness Centers")  (MH "Hospital Units+")  (MH "Hospitals+")  (MH "Practitioner's Office")  (MH "Rehabilitation Centers+")  (MH "Residential Facilities+")  (MH "Rural Health Centers")  (MH "Organizations")  (MH "Organizations, Nonprofit")  (MH "Professional Organizations") | (MH "Organizational Efficiency+")  (MH "Organizational Development")  (MH "Program Evaluation") |
| Textwords | "Action research"  "participatory research"  "participative research"  "collaborative inquiry"  "collaborative action"  "collaborative partnership*"  "participatory rural appraisal"  "participatory appraisal"  "emancipatory research"  "social reconnaissance"  "empowerment evaluation"  TI participatory AND (TI research OR TI design)  (TI action AND TI research)  "dialectical research"  "conscienti?ing research"  "participatory learning research"  "CBPR"   "community-based participatory research"   "community-based action research"  "participatory evaluation"  "participative evaluation"  "community-driven research"   "community-driven action research"  "action science"   "community-partnered action research"  "cooperative inquiry"  "dialectical inquiry"  "appreciative inquiry"  "decoloni?ing methodologies"  "democratic evaluation"  "recherche participative"  "recherche action"  "recherche collaborative" | organi?ation*  institution*  hospital  hospitals  facility  facilities  unit  units  ward  wards  clinic  clinics  pharmacy  pharmacies  Center  Centers  Centre  Centres  team  teams  agency  agencies  association  associations  "care trust”  “care trusts"  "healthcare trust”  “healthcare trusts"  "service* trust”  “service* trusts"  "hospital trust”  “hospital trusts" | ("process" AND ("Evaluation" OR "consultation" OR "apprais*"))  ("change" AND ("management" OR "implementation"))  (("management" OR "transformative") AND "research")  "community of practice"  "organi?ational learning"  "transformational change" |

# Database: PsycINFO

|  | **Concept: Action research** | **Concept: Organizational setting (facilities)** | **Concept: Organizational involvement / improvement** |
| --- | --- | --- | --- |
| Thesaurus Terms / Subheadings | exp Action Research/ | treatment facilities/  exp clinics/  exp community mental health centers/  exp hospitals/  exp nursing homes/  exp residential care institutions/  organizations/  nonprofit organizations/  professional organizations/   \|  \| \| --- \| | exp organizational learning/  exp "communities of practice"/  exp organizational behavior/  exp organizational change/  exp organizational characteristics/  exp organizational development/  exp organizational effectiveness/ |
| Textwords | "action research"  "participatory research"  "participative research"  "collaborative inquiry"  “collaborative action”  “collaborative partnership?”  "participatory rural appraisal"  "participatory appraisal"  "emancipatory research"  "social reconnaissance"  "empowerment evaluation"  ((research[title] OR design[title]) AND participatory[title])  (action[title] AND research[title])  “dialectical research”  “conscienti#ing research”  “participatory learning research”  “CBPR”  “community-based participatory research”  “community-based action research”  “participatory evaluation”  “participative evaluation”  “community-driven research”  “community-driven action research”  “action science”  “community-partnered action research”  “cooperative inquiry”  “dialectical inquiry”  “appreciative inquiry”  “decoloni#ing methodologies”  “democratic evaluation”  “recherche participative”  “recherché-action”  “recherche collaborative” | \|  \|  \| \| --- \| --- \|   organi#ation$.ti,ab.  institution$.ti,ab.  (hospital or hospitals).ti,ab.  (facility or facilities).ti,ab.  (unit or units).ti,ab.  (ward or wards).ti,ab.  (clinic or clinics).ti,ab.  (pharmacy or pharmacies).ti,ab.  (center or centers).ti,ab.  (centre or centres).ti,ab.  (team or teams).ti,ab.  (agency or agencies).ti,ab.  (association or associations).ti,ab.  (care trust or care trusts).ti,ab.  (healthcare trust or healthcare trusts).ti,ab.  (service? trust or service? trusts).ti,ab.  (hospital trust or hospital trusts).ti,ab. | (process[title] AND (evaluation[title] OR consultation[title] OR apprais*[title])) .ti,ab.  (change[title] AND (management[title] OR implementation[title]).ti,ab.  ((management[title] OR transformative[title]) AND research[title]).ti,ab.  (community ADJ1 practice).ti,ab.  (organi#ational learning).ti,ab.  transformational change.ti,ab. |

# Database: Social Work Abstracts

|  | **Concept: Action research** | **Concept: Organizational setting (facilities)** | **Concept: Organizational involvement / improvement** |
| --- | --- | --- | --- |
| Thesaurus Terms / Subheadings | action research.sh.  participatory research.sh.  collaborative research.sh. | clinics.sh.  hospitals.sh.  health centers.sh.  health organizations.sh.  health care organizations.sh. |  |
| Textwords | "action research"  "participatory research"  "participative research"  "collaborative inquiry"  “collaborative action”  “collaborative partnership?”  "participatory rural appraisal"  "participatory appraisal"  "emancipatory research"  "social reconnaissance"  "empowerment evaluation"  ((research[title] OR design[title]) AND participatory[title])  (action[title] AND research[title])  “dialectical research”  “conscienti#ing research”  “participatory learning research”  “CBPR”  “community-based participatory research”  “community-based action research”  “participatory evaluation”  “participative evaluation”  “community-driven research”  “community-driven action research”  “action science”  “community-partnered action research”  “cooperative inquiry”  “dialectical inquiry”  “appreciative inquiry”  “decoloni#ing methodologies”  “democratic evaluation”  “recherche participative”  “recherché-action”  “recherche collaborative” | \|  \|  \| \| --- \| --- \| |  |

# Database: Business Source Complete

|  | **Concept: Action research** | **Concept: Organizational setting (facilities)** | **Concept: Organizational involvement / improvement** |
| --- | --- | --- | --- |
| Thesaurus Terms / Subheadings |  | (DE "HOSPITALS")  (DE "CLINICS")  (DE "HEALTH facilities"  DE "HEALTH maintenance organizations"  DE "MEDICAL centers"  DE "HEALTH professionals' associations"   \|  \| \| --- \| \|  \| | DE "ORGANIZATIONAL change" OR DE "ORGANIZATIONAL effectiveness" |
| Textwords | "Action research"  "participatory research"  "participative research"  "collaborative inquiry"  "collaborative action"  "collaborative partnership*"  "participatory rural appraisal"  "participatory appraisal"  "emancipatory research"  "social reconnaissance"  "empowerment evaluation"  TI participatory AND (TI research OR TI design)  (TI action AND TI research) OR  "dialectical research"  "conscienti?ing research"  "participatory learning research"  "CBPR"   "community-based participatory research"   "community-based action research"  "participatory evaluation"  "participative evaluation"  "community-driven research"   "community-driven action research"  "action science"   "community-partnered action research"  "cooperative inquiry"  "dialectical inquiry"  "appreciative inquiry"  "decoloni?ing methodologies"  "democratic evaluation"  "recherche participative"  "recherche action"  "recherche collaborative" | \|  \|  \| \| --- \| --- \|   “health organi?ation*”  “healthcare organi?ation*”  “health institution*”  “healthcare institution”  hospital  hospitals  health facility  health facilities  health unit  health units  clinic  clinics  pharmacy  pharmacies  health Center  health Centers  health Centre  health Centres  health agency  health agencies  health association  health associations  "healthcare trust”  “healthcare trusts"  "health service* trust”  “health service* trusts"  "hospital trust”  “hospital trusts" | ("process" AND ("Evaluation" OR "consultation" OR "apprais*"))  ("change" AND ("management" OR "implementation"))  (("management" OR "transformative") AND "research")  "community of practice"  "organi?ational learning"  "transformational change" |

# Database: Cochrane library search strategy

| Search Name: | Full set 20121129 |  |
| --- | --- | --- |
| Date Run: | 29/11/12 21:12:55.655 |  |
|  |  |  |
|  |  |  |
| ID | Search | Hits |
| #1 | MeSH descriptor: [Community-Based Participatory Research] explode all trees | 53 |
| #2 | MeSH descriptor: [Community-Institutional Relations] explode all trees | 155 |
| #3 | MeSH descriptor: [Research] explode all trees | 11020 |
| #4 | research:ti (Word variations have been searched) | 5950 |
| #5 | #2 and (#3 or #4) | 33 |
| #6 | action research:ti,ab,kw | 127 |
| #7 | participatory research:ti,ab,kw | 100 |
| #8 | participative research:ti,ab,kw | 1 |
| #9 | collaborative inquiry:ti,ab,kw | 1 |
| #10 | collaborative action:ti,ab,kw | 1 |
| #11 | (collaborative next partnership*):ti,ab,kw | 10 |
| #12 | participatory rural appraisal:ti,ab,kw | 0 |
| #13 | participatory appraisal:ti,ab,kw | 0 |
| #14 | emancipatory research:ti,ab,kw | 0 |
| #15 | social reconnaissance:ti,ab,kw | 0 |
| #16 | empowerment evaluation:ti,ab,kw | 2 |
| #17 | (research:ti or design:ti) and participatory:ti | 35 |
| #18 | action:ti and research:ti | 42 |
| #19 | CBPR:ti,ab,kw | 21 |
| #20 | community based participatory research:ti,ab,kw | 87 |
| #21 | community based action research:ti,ab,kw | 1 |
| #22 | participatory evaluation:ti,ab,kw | 0 |
| #23 | community driven research:ti,ab,kw | 0 |
| #24 | action science:ti,ab,kw | 0 |
| #25 | cooperative inquiry:ti,ab,kw | 0 |
| #26 | appreciative inquiry:ti,ab,kw | 3 |
| #27 | democratic evaluation:ti,ab,kw | 0 |
| #28 | (decolono?ing next methodologies):ti,ab,kw | 0 |
| #29 | (conscienti?ing next research):ti,ab,kw | 0 |
| #30 | recherche action:ti,ab,kw | 2 |
| #31 | recherche participative:ti,ab,kw | 0 |
| #32 | recherche collaborative:ti,ab,kw | 0 |
| #33 | #1 or #5 or #6 or #7 or #8 or #9 or #10 or #11 or #12 or #13 or #14 or #15 or #16 or #17 or #18 or #19 or #20 or #21 or #22 or #23 or #24 or #25 or #26 or #27 or #28 or #29 or #30 or #31 or #32 | 292 |
| #34 | MeSH descriptor: [Residential Facilities] explode all trees | 1125 |
| #35 | MeSH descriptor: [Rehabilitation Centers] explode all trees | 505 |
| #36 | MeSH descriptor: [Physicians&apos; Offices] explode all trees | 30 |
| #37 | MeSH descriptor: [Pharmacies] explode all trees | 59 |
| #38 | MeSH descriptor: [Nurseries] explode all trees | 37 |
| #39 | MeSH descriptor: [Hospitals] explode all trees | 2420 |
| #40 | MeSH descriptor: [Hospital Units] explode all trees | 2554 |
| #41 | MeSH descriptor: [Fitness Centers] explode all trees | 19 |
| #42 | MeSH descriptor: [Dental Facilities] explode all trees | 54 |
| #43 | MeSH descriptor: [Birthing Centers] explode all trees | 19 |
| #44 | MeSH descriptor: [Ambulatory Care Facilities] explode all trees | 1430 |
| #45 | MeSH descriptor: [Academic Medical Centers] explode all trees | 1109 |
| #46 | MeSH descriptor: [Health Facilities] this term only | 34 |
| #47 | MeSH descriptor: [Organizations] this term only | 8 |
| #48 | MeSH descriptor: [Organizations, Nonprofit] this term only | 11 |
| #49 | organi?ation*:ti,ab | 4946 |
| #50 | institution*:ti,ab | 5489 |
| #51 | hospital:ti,ab | 36342 |
| #52 | facility:ti,ab | 2918 |
| #53 | unit:ti,ab | 19013 |
| #54 | ward:ti,ab | 2493 |
| #55 | clinic:ti,ab | 12735 |
| #56 | pharmacy:ti,ab | 968 |
| #57 | center:ti,ab | 29530 |
| #58 | team:ti,ab | 3073 |
| #59 | agency:ti,ab | 947 |
| #60 | association:ti,ab | 18689 |
| #61 | (care next trust*):ti,ab | 52 |
| #62 | (healthcare next trust*):ti,ab | 6 |
| #63 | (service* next trust*):ti,ab | 13 |
| #64 | (hospital next trust*):ti,ab | 24 |
| #65 | #34 or #35 or #36 or #37 or #38 or #39 or #40 or #41 or #42 or #43 or #44 or #45 or #46 or #47 or #48 or #49 or #50 or #51 or #52 or #53 or #54 or #55 or #56 or #57 or #58 or #59 or #60 or #61 or #62 or #63 or #64 | 114273 |
| #66 | MeSH descriptor: [Organizational Innovation] this term only | 68 |
| #67 | (evaluation:ti,ab or consultation:ti,ab) and process:ti,ab | 2298 |
| #68 | (management:ti,ab or implementation:ti,ab) and change:ti,ab | 5781 |
| #69 | (management:ti,ab or transformative:ti,ab) and research:ti,ab | 2868 |
| #70 | community or practice:ti,ab | 0 |
| #71 | (organi?ational next learning):ti,ab | 1 |
| #72 | transformational change:ti,ab | 0 |
| #73 | #66 or #67 or #68 or #69 or #70 or #71 or #72 | 10096 |
| #74 | #33 and (#65 or #73) (Word variations have been searched) | 133 |

# Database: PubMed search strategy

| **Search** | **Add to builder** | **Query** | **Items found** |
| --- | --- | --- | --- |
| [#103](http://www.ncbi.nlm.nih.gov/pubmed/advanced) | [Add](http://www.ncbi.nlm.nih.gov/pubmed/advanced) | Search **(#58) AND #100** Filters: **French; English** | 3336 |
| [#102](http://www.ncbi.nlm.nih.gov/pubmed/advanced) | [Add](http://www.ncbi.nlm.nih.gov/pubmed/advanced) | Search **(#58) AND #100** Filters: **French** | [33](http://www.ncbi.nlm.nih.gov/pubmed/?cmd=HistorySearch&querykey=102) |
| [#101](http://www.ncbi.nlm.nih.gov/pubmed/advanced) | [Add](http://www.ncbi.nlm.nih.gov/pubmed/advanced) | Search **(#58) AND #100** | [3414](http://www.ncbi.nlm.nih.gov/pubmed/?cmd=HistorySearch&querykey=101) |
| [#100](http://www.ncbi.nlm.nih.gov/pubmed/advanced) | [Add](http://www.ncbi.nlm.nih.gov/pubmed/advanced) | Search **(#91) OR #99** | [2753193](http://www.ncbi.nlm.nih.gov/pubmed/?cmd=HistorySearch&querykey=100) |
| [#99](http://www.ncbi.nlm.nih.gov/pubmed/advanced) | [Add](http://www.ncbi.nlm.nih.gov/pubmed/advanced) | Search **((((((#92) OR #93) OR #94) OR #95) OR #96) OR #97) OR #98** | [132155](http://www.ncbi.nlm.nih.gov/pubmed/?cmd=HistorySearch&querykey=99) |
| [#98](http://www.ncbi.nlm.nih.gov/pubmed/advanced) | [Add](http://www.ncbi.nlm.nih.gov/pubmed/advanced) | Search **"transformational change"[tiab]** | [109](http://www.ncbi.nlm.nih.gov/pubmed/?cmd=HistorySearch&querykey=98) |
| [#97](http://www.ncbi.nlm.nih.gov/pubmed/advanced) | [Add](http://www.ncbi.nlm.nih.gov/pubmed/advanced) | Search **"organizational learning"[tiab] OR "organisational learning"[tiab]** | [235](http://www.ncbi.nlm.nih.gov/pubmed/?cmd=HistorySearch&querykey=97) |
| [#96](http://www.ncbi.nlm.nih.gov/pubmed/advanced) | [Add](http://www.ncbi.nlm.nih.gov/pubmed/advanced) | Search **"community of practice"[tiab]** | [149](http://www.ncbi.nlm.nih.gov/pubmed/?cmd=HistorySearch&querykey=96) |
| [#95](http://www.ncbi.nlm.nih.gov/pubmed/advanced) | [Add](http://www.ncbi.nlm.nih.gov/pubmed/advanced) | Search **((management[tiab] OR transformative[tiab]) AND research[tiab])** | [46386](http://www.ncbi.nlm.nih.gov/pubmed/?cmd=HistorySearch&querykey=95) |
| [#94](http://www.ncbi.nlm.nih.gov/pubmed/advanced) | [Add](http://www.ncbi.nlm.nih.gov/pubmed/advanced) | Search **((management[tiab] OR implementation[tiab]) AND change[tiab])** | [31467](http://www.ncbi.nlm.nih.gov/pubmed/?cmd=HistorySearch&querykey=94) |
| [#93](http://www.ncbi.nlm.nih.gov/pubmed/advanced) | [Add](http://www.ncbi.nlm.nih.gov/pubmed/advanced) | Search **((evaluation[tiab] OR consultation[tiab]) AND process[tiab])** | [39594](http://www.ncbi.nlm.nih.gov/pubmed/?cmd=HistorySearch&querykey=93) |
| [#92](http://www.ncbi.nlm.nih.gov/pubmed/advanced) | [Add](http://www.ncbi.nlm.nih.gov/pubmed/advanced) | Search **organizational innovation[MeSH Terms]** | [21165](http://www.ncbi.nlm.nih.gov/pubmed/?cmd=HistorySearch&querykey=92) |
| [#91](http://www.ncbi.nlm.nih.gov/pubmed/advanced) | [Add](http://www.ncbi.nlm.nih.gov/pubmed/advanced) | Search **((((((((((((((((((((((((((((((#59) OR #60) OR #61) OR #62) OR #63) OR #64) OR #65) OR #66) OR #67) OR #68) OR #69) OR #70) OR #71) OR #72) OR #73) OR #75) OR #76) OR #77) OR #78) OR #79) OR #80) OR #81) OR #82) OR #83) OR #84) OR #85) OR #86) OR #87) OR #88) OR #89) OR #90** | [2672925](http://www.ncbi.nlm.nih.gov/pubmed/?cmd=HistorySearch&querykey=91) |
| [#90](http://www.ncbi.nlm.nih.gov/pubmed/advanced) | [Add](http://www.ncbi.nlm.nih.gov/pubmed/advanced) | Search **"hospital trust"[tiab] OR "hospital trusts"[tiab]** | [449](http://www.ncbi.nlm.nih.gov/pubmed/?cmd=HistorySearch&querykey=90) |
| [#89](http://www.ncbi.nlm.nih.gov/pubmed/advanced) | [Add](http://www.ncbi.nlm.nih.gov/pubmed/advanced) | Search **"service trust"[tiab] OR "service trusts"[tiab] OR "services trust"[tiab] OR "services trusts"[tiab]** | [236](http://www.ncbi.nlm.nih.gov/pubmed/?cmd=HistorySearch&querykey=89) |
| [#88](http://www.ncbi.nlm.nih.gov/pubmed/advanced) | [Add](http://www.ncbi.nlm.nih.gov/pubmed/advanced) | Search **"healthcare trust"[tiab] OR "healthcare trusts"[tiab]** | [47](http://www.ncbi.nlm.nih.gov/pubmed/?cmd=HistorySearch&querykey=88) |
| [#87](http://www.ncbi.nlm.nih.gov/pubmed/advanced) | [Add](http://www.ncbi.nlm.nih.gov/pubmed/advanced) | Search **"care trust"[tiab] OR "care trusts"[tiab]** | [1165](http://www.ncbi.nlm.nih.gov/pubmed/?cmd=HistorySearch&querykey=87) |
| [#86](http://www.ncbi.nlm.nih.gov/pubmed/advanced) | [Add](http://www.ncbi.nlm.nih.gov/pubmed/advanced) | Search **association[tiab] OR associations[tiab]** | [690052](http://www.ncbi.nlm.nih.gov/pubmed/?cmd=HistorySearch&querykey=86) |
| [#85](http://www.ncbi.nlm.nih.gov/pubmed/advanced) | [Add](http://www.ncbi.nlm.nih.gov/pubmed/advanced) | Search **agency[tiab] OR agencies[tiab]** | [46983](http://www.ncbi.nlm.nih.gov/pubmed/?cmd=HistorySearch&querykey=85) |
| [#84](http://www.ncbi.nlm.nih.gov/pubmed/advanced) | [Add](http://www.ncbi.nlm.nih.gov/pubmed/advanced) | Search **team[tiab] OR teams[tiab]** | [78047](http://www.ncbi.nlm.nih.gov/pubmed/?cmd=HistorySearch&querykey=84) |
| [#83](http://www.ncbi.nlm.nih.gov/pubmed/advanced) | [Add](http://www.ncbi.nlm.nih.gov/pubmed/advanced) | Search **center[tiab] OR centers[tiab] OR centre[tiab] OR centres[tiab]** | [436102](http://www.ncbi.nlm.nih.gov/pubmed/?cmd=HistorySearch&querykey=83) |
| [#82](http://www.ncbi.nlm.nih.gov/pubmed/advanced) | [Add](http://www.ncbi.nlm.nih.gov/pubmed/advanced) | Search **pharmacy[tiab] OR pharmacies[tiab]** | [26218](http://www.ncbi.nlm.nih.gov/pubmed/?cmd=HistorySearch&querykey=82) |
| [#81](http://www.ncbi.nlm.nih.gov/pubmed/advanced) | [Add](http://www.ncbi.nlm.nih.gov/pubmed/advanced) | Search **clinic[tiab] OR clinics[tiab]** | [181866](http://www.ncbi.nlm.nih.gov/pubmed/?cmd=HistorySearch&querykey=81) |
| [#80](http://www.ncbi.nlm.nih.gov/pubmed/advanced) | [Add](http://www.ncbi.nlm.nih.gov/pubmed/advanced) | Search **ward[tiab] OR wards[tiab]** | [35988](http://www.ncbi.nlm.nih.gov/pubmed/?cmd=HistorySearch&querykey=80) |
| [#79](http://www.ncbi.nlm.nih.gov/pubmed/advanced) | [Add](http://www.ncbi.nlm.nih.gov/pubmed/advanced) | Search **unit[tiab] OR units[tiab]** | [399873](http://www.ncbi.nlm.nih.gov/pubmed/?cmd=HistorySearch&querykey=79) |
| [#78](http://www.ncbi.nlm.nih.gov/pubmed/advanced) | [Add](http://www.ncbi.nlm.nih.gov/pubmed/advanced) | Search **facility[tiab] OR facilities[tiab]** | [84909](http://www.ncbi.nlm.nih.gov/pubmed/?cmd=HistorySearch&querykey=78) |
| [#77](http://www.ncbi.nlm.nih.gov/pubmed/advanced) | [Add](http://www.ncbi.nlm.nih.gov/pubmed/advanced) | Search **hospital[tiab] OR hospitals[tiab]** | [665883](http://www.ncbi.nlm.nih.gov/pubmed/?cmd=HistorySearch&querykey=77) |
| [#76](http://www.ncbi.nlm.nih.gov/pubmed/advanced) | [Add](http://www.ncbi.nlm.nih.gov/pubmed/advanced) | Search **institution[tiab] OR institutions[tiab] OR institutional[tiab]** | [136739](http://www.ncbi.nlm.nih.gov/pubmed/?cmd=HistorySearch&querykey=76) |
| [#75](http://www.ncbi.nlm.nih.gov/pubmed/advanced) | [Add](http://www.ncbi.nlm.nih.gov/pubmed/advanced) | Search **organisation[tiab] OR organisations[tiab] OR organisational[tiab] OR organization[tiab] OR organizations[tiab] OR organizational[tiab]** | [253800](http://www.ncbi.nlm.nih.gov/pubmed/?cmd=HistorySearch&querykey=75) |
| [#73](http://www.ncbi.nlm.nih.gov/pubmed/advanced) | [Add](http://www.ncbi.nlm.nih.gov/pubmed/advanced) | Search **organizations,Nonprofit[MeSH:noexp]** | [2574](http://www.ncbi.nlm.nih.gov/pubmed/?cmd=HistorySearch&querykey=73) |
| [#72](http://www.ncbi.nlm.nih.gov/pubmed/advanced) | [Add](http://www.ncbi.nlm.nih.gov/pubmed/advanced) | Search **organizations[MeSH:noexp]** | [6080](http://www.ncbi.nlm.nih.gov/pubmed/?cmd=HistorySearch&querykey=72) |
| [#71](http://www.ncbi.nlm.nih.gov/pubmed/advanced) | [Add](http://www.ncbi.nlm.nih.gov/pubmed/advanced) | Search **health facilities[MeSH:noexp]** | [10797](http://www.ncbi.nlm.nih.gov/pubmed/?cmd=HistorySearch&querykey=71) |
| [#70](http://www.ncbi.nlm.nih.gov/pubmed/advanced) | [Add](http://www.ncbi.nlm.nih.gov/pubmed/advanced) | Search **academic medical centers[MeSH Terms]** | [65009](http://www.ncbi.nlm.nih.gov/pubmed/?cmd=HistorySearch&querykey=70) |
| [#69](http://www.ncbi.nlm.nih.gov/pubmed/advanced) | [Add](http://www.ncbi.nlm.nih.gov/pubmed/advanced) | Search **ambulatory care facilities[MeSH Terms]** | [40857](http://www.ncbi.nlm.nih.gov/pubmed/?cmd=HistorySearch&querykey=69) |
| [#68](http://www.ncbi.nlm.nih.gov/pubmed/advanced) | [Add](http://www.ncbi.nlm.nih.gov/pubmed/advanced) | Search **birthing centers[MeSH Terms]** | [486](http://www.ncbi.nlm.nih.gov/pubmed/?cmd=HistorySearch&querykey=68) |
| [#67](http://www.ncbi.nlm.nih.gov/pubmed/advanced) | [Add](http://www.ncbi.nlm.nih.gov/pubmed/advanced) | Search **dental facilities[MeSH Terms]** | [7564](http://www.ncbi.nlm.nih.gov/pubmed/?cmd=HistorySearch&querykey=67) |
| [#66](http://www.ncbi.nlm.nih.gov/pubmed/advanced) | [Add](http://www.ncbi.nlm.nih.gov/pubmed/advanced) | Search **fitness centers[MeSH Terms]** | [298](http://www.ncbi.nlm.nih.gov/pubmed/?cmd=HistorySearch&querykey=66) |
| [#65](http://www.ncbi.nlm.nih.gov/pubmed/advanced) | [Add](http://www.ncbi.nlm.nih.gov/pubmed/advanced) | Search **hospital units[MeSH Terms]** | [70715](http://www.ncbi.nlm.nih.gov/pubmed/?cmd=HistorySearch&querykey=65) |
| [#64](http://www.ncbi.nlm.nih.gov/pubmed/advanced) | [Add](http://www.ncbi.nlm.nih.gov/pubmed/advanced) | Search **hospitals[MeSH Terms]** | [187603](http://www.ncbi.nlm.nih.gov/pubmed/?cmd=HistorySearch&querykey=64) |
| [#63](http://www.ncbi.nlm.nih.gov/pubmed/advanced) | [Add](http://www.ncbi.nlm.nih.gov/pubmed/advanced) | Search **nurseries[MeSH Terms]** | [1943](http://www.ncbi.nlm.nih.gov/pubmed/?cmd=HistorySearch&querykey=63) |
| [#62](http://www.ncbi.nlm.nih.gov/pubmed/advanced) | [Add](http://www.ncbi.nlm.nih.gov/pubmed/advanced) | Search **pharmacies[MeSH Terms]** | [3571](http://www.ncbi.nlm.nih.gov/pubmed/?cmd=HistorySearch&querykey=62) |
| [#61](http://www.ncbi.nlm.nih.gov/pubmed/advanced) | [Add](http://www.ncbi.nlm.nih.gov/pubmed/advanced) | Search **physician offices[MeSH Terms]** | [1404](http://www.ncbi.nlm.nih.gov/pubmed/?cmd=HistorySearch&querykey=61) |
| [#60](http://www.ncbi.nlm.nih.gov/pubmed/advanced) | [Add](http://www.ncbi.nlm.nih.gov/pubmed/advanced) | Search **rehabilitation centers[MeSH Terms]** | [11199](http://www.ncbi.nlm.nih.gov/pubmed/?cmd=HistorySearch&querykey=60) |
| [#59](http://www.ncbi.nlm.nih.gov/pubmed/advanced) | [Add](http://www.ncbi.nlm.nih.gov/pubmed/advanced) | Search **residential facilities[MeSH Terms]** | [40661](http://www.ncbi.nlm.nih.gov/pubmed/?cmd=HistorySearch&querykey=59) |
| [#58](http://www.ncbi.nlm.nih.gov/pubmed/advanced) | [Add](http://www.ncbi.nlm.nih.gov/pubmed/advanced) | Search **(((((((((((((((((((((((((#32) OR #33) OR #34) OR #35) OR #36) OR #37) OR #38) OR #39) OR #40) OR #41) OR #42) OR #43) OR #44) OR #45) OR #46) OR #47) OR #48) OR #49) OR #50) OR #51) OR #52) OR #53) OR #54) OR #55) OR #56) OR #57** | [7346](http://www.ncbi.nlm.nih.gov/pubmed/?cmd=HistorySearch&querykey=58) |
| [#57](http://www.ncbi.nlm.nih.gov/pubmed/advanced) | [Add](http://www.ncbi.nlm.nih.gov/pubmed/advanced) | Search **"recherche action"[tw]** | [1](http://www.ncbi.nlm.nih.gov/pubmed/?cmd=HistorySearch&querykey=57) |
| [#56](http://www.ncbi.nlm.nih.gov/pubmed/advanced) | [Add](http://www.ncbi.nlm.nih.gov/pubmed/advanced) | Search **"democratic evaluation"[tw]** | [4](http://www.ncbi.nlm.nih.gov/pubmed/?cmd=HistorySearch&querykey=56) |
| [#55](http://www.ncbi.nlm.nih.gov/pubmed/advanced) | [Add](http://www.ncbi.nlm.nih.gov/pubmed/advanced) | Search **"appreciative inquiry"[tw]** | [85](http://www.ncbi.nlm.nih.gov/pubmed/?cmd=HistorySearch&querykey=55) |
| [#54](http://www.ncbi.nlm.nih.gov/pubmed/advanced) | [Add](http://www.ncbi.nlm.nih.gov/pubmed/advanced) | Search **"cooperative inquiry"[tw]** | [24](http://www.ncbi.nlm.nih.gov/pubmed/?cmd=HistorySearch&querykey=54) |
| [#53](http://www.ncbi.nlm.nih.gov/pubmed/advanced) | [Add](http://www.ncbi.nlm.nih.gov/pubmed/advanced) | Search **"action science"[tw]** | [14](http://www.ncbi.nlm.nih.gov/pubmed/?cmd=HistorySearch&querykey=53) |
| [#52](http://www.ncbi.nlm.nih.gov/pubmed/advanced) | [Add](http://www.ncbi.nlm.nih.gov/pubmed/advanced) | Search **"community driven research"[tw]** | [14](http://www.ncbi.nlm.nih.gov/pubmed/?cmd=HistorySearch&querykey=52) |
| [#51](http://www.ncbi.nlm.nih.gov/pubmed/advanced) | [Add](http://www.ncbi.nlm.nih.gov/pubmed/advanced) | Search **"participatory evaluation"[tw]** | [87](http://www.ncbi.nlm.nih.gov/pubmed/?cmd=HistorySearch&querykey=51) |
| [#50](http://www.ncbi.nlm.nih.gov/pubmed/advanced) | [Add](http://www.ncbi.nlm.nih.gov/pubmed/advanced) | Search **"community based action research"[tw]** | [14](http://www.ncbi.nlm.nih.gov/pubmed/?cmd=HistorySearch&querykey=50) |
| [#49](http://www.ncbi.nlm.nih.gov/pubmed/advanced) | [Add](http://www.ncbi.nlm.nih.gov/pubmed/advanced) | Search **"community based participatory research"[tw]** | [1732](http://www.ncbi.nlm.nih.gov/pubmed/?cmd=HistorySearch&querykey=49) |
| [#48](http://www.ncbi.nlm.nih.gov/pubmed/advanced) | [Add](http://www.ncbi.nlm.nih.gov/pubmed/advanced) | Search **CBPR[tw]** | [427](http://www.ncbi.nlm.nih.gov/pubmed/?cmd=HistorySearch&querykey=48) |
| [#47](http://www.ncbi.nlm.nih.gov/pubmed/advanced) | [Add](http://www.ncbi.nlm.nih.gov/pubmed/advanced) | Search **(action[Title] AND research[Title])** | [2167](http://www.ncbi.nlm.nih.gov/pubmed/?cmd=HistorySearch&querykey=47) |
| [#46](http://www.ncbi.nlm.nih.gov/pubmed/advanced) | [Add](http://www.ncbi.nlm.nih.gov/pubmed/advanced) | Search **(participatory[Title] AND (research[Title] OR design[Title]))** | [741](http://www.ncbi.nlm.nih.gov/pubmed/?cmd=HistorySearch&querykey=46) |
| [#45](http://www.ncbi.nlm.nih.gov/pubmed/advanced) | [Add](http://www.ncbi.nlm.nih.gov/pubmed/advanced) | Search **"empowerment evaluation"[tw]** | [20](http://www.ncbi.nlm.nih.gov/pubmed/?cmd=HistorySearch&querykey=45) |
| [#44](http://www.ncbi.nlm.nih.gov/pubmed/advanced) | [Add](http://www.ncbi.nlm.nih.gov/pubmed/advanced) | Search **"social reconnaissance"[tw]** | [2](http://www.ncbi.nlm.nih.gov/pubmed/?cmd=HistorySearch&querykey=44) |
| [#43](http://www.ncbi.nlm.nih.gov/pubmed/advanced) | [Add](http://www.ncbi.nlm.nih.gov/pubmed/advanced) | Search **"emancipatory research"[tw]** | [8](http://www.ncbi.nlm.nih.gov/pubmed/?cmd=HistorySearch&querykey=43) |
| [#42](http://www.ncbi.nlm.nih.gov/pubmed/advanced) | [Add](http://www.ncbi.nlm.nih.gov/pubmed/advanced) | Search **"participatory appraisal"[tw]** | [29](http://www.ncbi.nlm.nih.gov/pubmed/?cmd=HistorySearch&querykey=42) |
| [#41](http://www.ncbi.nlm.nih.gov/pubmed/advanced) | [Add](http://www.ncbi.nlm.nih.gov/pubmed/advanced) | Search **"participatory rural appraisal"[tw]** | [60](http://www.ncbi.nlm.nih.gov/pubmed/?cmd=HistorySearch&querykey=41) |
| [#40](http://www.ncbi.nlm.nih.gov/pubmed/advanced) | [Add](http://www.ncbi.nlm.nih.gov/pubmed/advanced) | Search **"collaborative partnerships"[tw]** | [159](http://www.ncbi.nlm.nih.gov/pubmed/?cmd=HistorySearch&querykey=40) |
| [#39](http://www.ncbi.nlm.nih.gov/pubmed/advanced) | [Add](http://www.ncbi.nlm.nih.gov/pubmed/advanced) | Search **"collaborative partnership"[tw]** | [185](http://www.ncbi.nlm.nih.gov/pubmed/?cmd=HistorySearch&querykey=39) |
| [#38](http://www.ncbi.nlm.nih.gov/pubmed/advanced) | [Add](http://www.ncbi.nlm.nih.gov/pubmed/advanced) | Search **"collaborative action"[tw]** | [116](http://www.ncbi.nlm.nih.gov/pubmed/?cmd=HistorySearch&querykey=38) |
| [#37](http://www.ncbi.nlm.nih.gov/pubmed/advanced) | [Add](http://www.ncbi.nlm.nih.gov/pubmed/advanced) | Search **"collaborative inquiry"[tw]** | [26](http://www.ncbi.nlm.nih.gov/pubmed/?cmd=HistorySearch&querykey=37) |
| [#36](http://www.ncbi.nlm.nih.gov/pubmed/advanced) | [Add](http://www.ncbi.nlm.nih.gov/pubmed/advanced) | Search **"participative research"[tw]** | [15](http://www.ncbi.nlm.nih.gov/pubmed/?cmd=HistorySearch&querykey=36) |
| [#35](http://www.ncbi.nlm.nih.gov/pubmed/advanced) | [Add](http://www.ncbi.nlm.nih.gov/pubmed/advanced) | Search **"participatory research"[tw]** | [2135](http://www.ncbi.nlm.nih.gov/pubmed/?cmd=HistorySearch&querykey=35) |
| [#34](http://www.ncbi.nlm.nih.gov/pubmed/advanced) | [Add](http://www.ncbi.nlm.nih.gov/pubmed/advanced) | Search **"action research"[tw]** | [2112](http://www.ncbi.nlm.nih.gov/pubmed/?cmd=HistorySearch&querykey=34) |
| [#33](http://www.ncbi.nlm.nih.gov/pubmed/advanced) | [Add](http://www.ncbi.nlm.nih.gov/pubmed/advanced) | Search **(community institutional relations[MeSH Terms] AND (research[Title] OR research[MeSH Terms]))** | [1120](http://www.ncbi.nlm.nih.gov/pubmed/?cmd=HistorySearch&querykey=33) |
| [#32](http://www.ncbi.nlm.nih.gov/pubmed/advanced) | [Add](http://www.ncbi.nlm.nih.gov/pubmed/advanced) | Search **community based participatory research[MeSH Terms]** | [1242](http://www.ncbi.nlm.nih.gov/pubmed/?cmd=HistorySearch&querykey=32) |

# Grey literature search strategy

We attempted to develop a structured approach to searching grey literature that could be replicated in the future. We decided not to conduct hand searching.

| **Grey literature sources (database, organization, website)** | **Date searched** | **URL** | **Search terms** | **Number of all hits** | **Number of relevant documents out of all scanned** | **Notes / Observations** |
| --- | --- | --- | --- | --- | --- | --- |
| Google (advanced) | 2013-03-14 |  | allintitle: ("action research" OR "participatory research" OR " community based action research") (hospital OR "care facility" OR clinic OR "health trust" OR ward OR pharmacy OR nurses OR physician OR therapist) Notes: no language limits, ranked by relevance | 531 | Two raters scanned and reached consensus on 50 hits (9.4% of all hits, not blind rating). 11 documents included based on eligibility criteria. | Out of the scanned 50 hits, 23 were duplicates with documents already included in our sample from searching in bibliographic databases. The search was limited to title only in order to have a more manageable sample. Therefore, most of the retrieved hits were journal articles or theses. |
| The New York academy of Medicine, Grey Literature Report | 2013-03-14 | http://www.greylit.org/ | “participatory research” Search field: full text, Search all reports, | 8 | Two raters scanned and reached consensus on all 8 hits (not blind). All 8 were excluded. | The retrieved hits dealt with community-based participatory research. |
| OpenGrey | 2013-03-14 | http://www.opengrey.eu/ | ("action research" OR "participatory research" OR " community based action research") AND (hospital OR "care facility" OR clinic OR  "health trust" OR ward OR pharmacy OR nurses OR physician OR therapist) | 4 | Two rates scanned and reached consensus on all 4 hits. Two were included. | Judgement was made based on title and provided keywords, as abstract or full text were not available. |
| OpenGrey | 2013-03-14 | http://www.opengrey.eu/ | "action research" discipline:(05T - Health services, health administration, community care services) | 33 | Two rates scanned and reached consensus on all 33 hits. 10 were included. | Same as above |
| ProQuest Dissertations & Theses: UK & Ireland: Health & Medicine, ProQuest Dissertations & Theses Full Text: Health & Medicine | 2013-03-15 | http://search.proquest.com/health | su.Exact("action research" OR "participatory research") Manuscript type: Doctoral dissertations, Master's theses  Language: English, French | 95 |  | The core research group decided to limit the search for theses and dissertation to subject headings only (not searching with keywords). |

Process for identification of grey literature: The two raters (PB and VG) scanned all hits (titles and abstracts when available) simultaneously / in real time, mostly since the content (such as grey literature found through Google is constantly changing). Through discussion (not blind process), they reached agreement. They used pre-established eligibility criteria for identification of potentially relevant hits. Since these two raters have been coding (independently) to identify (nearly 9000 research abstracts) and select (125 full text articles) potentially relevant studies using established eligibility criteria, they have a high degree of agreement and coherence. In scanning grey literature resources, they looked for evidence of organizational change in projects where health organizations were involved in participatory research.
